# SwimmingIndividuals: A high-performance agent-based model for marine ecosystems

**DOI:** 10.1371/journal.pcbi.1013610

**Published:** 2026-11-06

**Authors:** Matthew S. Woodstock, Jann Paul Mattern, Zhen Wu, Gregory L. Britten

**Affiliations:** 1 Cooperative Institute for Marine and Atmospheric Studies, Rosenstiel School of Marine, Atmospheric, and Earth Science, University of Miami, Miami, Florida, United States of America; 2 Woods Hole Oceanographic Institution, Woods Hole, Massachusetts, United States of America; 3 NOAA Southeast Fisheries Science Center, Miami, Florida, United States of America; 4 University of California Santa Cruz, Santa Cruz, California, United States of America; 5 College of Environmental Sciences and Engineering, Peking University, Beijing, China; Chinese Academy of Sciences, CHINA

## Abstract

Because they are based on established theory, mechanistic models are essential for projecting ecological responses to novel conditions. Yet mechanistic agent-based model (ABM) applications have often been limited by computational constraints. This paper introduces *SwimmingIndividuals*, an open-source ABM framework that advances our ability to simulate physiological processes, animal movement, higher trophic level marine ecosystem dynamics, and fisheries removals at large ecological scales. The software's key contributions are a suite of mechanistic sub-models governing the full life cycle of each agent, including visual prey detection, adaptive behaviors such as diel vertical migration (DVM), a flexible bioenergetics engine with three taxon-specific metabolic architectures, and a spatial pathfinding algorithm for habitat-seeking movement. These processes are made computationally tractable through a hybrid CPU/GPU architecture written in Julia. Unlike prior ABMs that use fixed perception radii or single-equation bioenergetics, *SwimmingIndividuals* enables emergent ecological dynamics across populations of superindividual agents. We demonstrate the framework through three Gulf of America simulations. The model reproduced DVM, cetacean diving patterns, size-based trophic shifts, and von Bertalanffy growth curves consistent with published King Mackerel stock assessments. Long-term simulations generated realistic population dynamics and quantified impacts of fishery harvest control rules across five fleets. *SwimmingIndividuals* provides a powerful, flexible, open-source platform for mechanistic marine ecosystem research and ecosystem-based fisheries management.

## Author summary

To understand and predict how marine ecosystems function, we need to account for the decisions and life histories of the individual animals within them. We developed *SwimmingIndividuals*, an open-source ‘virtual laboratory’ for marine ecology and fisheries science. This software creates large, realistic simulations of virtual organisms from multiple taxa simultaneously, each with unique biological traits. Modeled animals make decisions based on their internal physiological state and their perception of light, temperature, and prey. By simulating these individual actions at scale, complex ecosystem-level patterns (e.g., population dynamics, food web structure, and seasonal migrations) emerge without being explicitly programmed. *SwimmingIndividuals* provides the scientific community with a tool to investigate fundamental biological questions, explore ‘what-if’ scenarios for fisheries management, and forecast how marine life can respond to future environmental changes.

## Introduction

Predicting the dynamics of marine ecosystems is a critical challenge, as the cumulative effect of individual-scale processes produces emergent, often non-linear dynamics at population and community scales [1]. Capturing this complexity is essential for conservation and management, particularly under the co-occurring stressors from climate change and fishing [2]. Agent-based models (ABMs) represent a fine-scale approach to these questions, modeling individuals as discrete, autonomous entities with unique state variables and behavioral rules [3]. This mechanistic approach is powerful because population-level responses (e.g., shifts in distribution, recruitment) arise directly from individuals responding to their local environment based on physiological and behavioral principles [4,5].

Traditional ecological models often rely on fitting statistical relationships to historical data, an approach that is inherently limited in mechanistic explanatory power and sensitive to misspecification when extrapolating into novel conditions [6,7]. Although ABMs are better suited for such projections, computational limitations have historically restricted them to smaller spatial scales and fewer agents [8]. These constraints force simplified rules for key processes: an agent's perception may be a fixed radius rather than being mechanistically determined by light and physiology [9], or its bioenergetics may not be flexible enough to capture diverse metabolic and behavioral strategies [10].

To address these limitations, we developed *SwimmingIndividuals*, a mechanistic agent-based model that exploits modern high-performance computing to integrate ABM processes into an ecosystem modeling framework. *SwimmingIndividuals* couples a physics-based visual detection model derived from light attenuation theory with a flexible, multi-taxon bioenergetics engine and a GPU-accelerated spatial architecture. The result is a framework capable of simulating the full life histories of superindividual agents (i.e., a collection of like-sized organisms with the same traits) across a three-dimensional marine domain. We describe *SwimmingIndividuals*, its governing equations and architecture in full and explain contrast with the closest existing frameworks. In this paper, we describe the full mathematical structure and software architecture of *SwimmingIndividuals* and demonstrate its ability to reproduce complex ecological phenomena through a series of worked examples in the Gulf of America, providing a flexible, open-source tool for testing fundamental ecological theory and exploring applied management strategies.

## Design and implementation

### Overview and design concepts

*SwimmingIndividuals* simulates the behavior, interactions, movement, and bioenergetics of multiple marine species within a dynamic, spatially explicit environment. The model consists of four primary entities: focal species agents, gridded resource patches, a dynamic abiotic environment, and fisheries harvesting (Fig 1).

**Fig 1:**
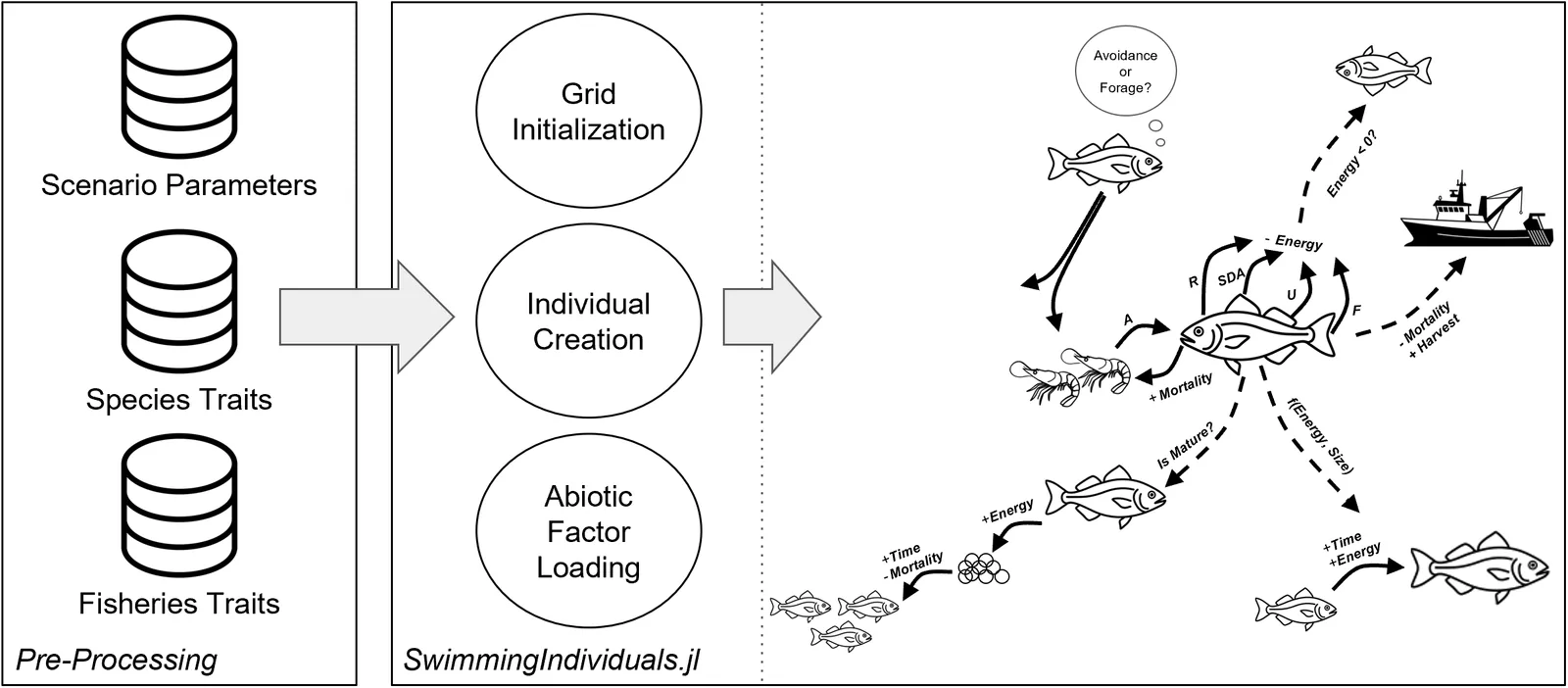
Schematic of the *SwimmingIndividuals* model, showing the agent configuration (traits) required, the initial steps that create the simulated ecosystem, and the behavioral, bioenergetic, and fisheries processes that govern the simulation. A = assimilated energy, R = respired energy, SDA = specific dynamic action, U = egested energy, F = excreted energy. All schematic icons in Fig 1 were created by the authors and contain no third-party copyrighted elements.

The four primary entities interact as follows. Focal species agents are the principal objects of simulation: each agent represents a superindividual with individual state variables for length, mass, gut fullness, energy reserves, maturity status, depth, and geographic position. Resource patches are spatially explicit grids of biomass representing key predator, prey, and competitor populations too numerous to resolve individually; they are subject to logistic growth, seasonal redistribution, and depletion by agent predation. The abiotic environment is a set of monthly three-dimensional raster fields (e.g., temperature, salinity, bathymetric depth) supplied as a time-varying series of ESRI ASCII (.asc) rasters catalogued by a XML configuration and updated at each monthly transition; when the simulation extends beyond the last available month, the final year of layers is replayed so the seasonal cycle continues. Fisheries are fleet objects defined by regulatory parameters (season, area, quota, gear selectivity) that interact with agents through probabilistic biomass removal at each timestep.

Superindividual agents include state variables relevant at both the individual and agent scales [11]. Each agent carries an *abundance* field representing the number of individuals in the agent along with two biomass fields: *biomass*_*ind*_ (individual biomass, g) and *biomass*_*school*_ (total agent biomass, g). Since all individuals in a superindividual are identical, *biomass*_*school*_ is equivalent to the product of *biomass*_*ind*_ and *abundance*, but both fields are necessary for the accurate and efficient computation of both individual-based (e.g., respiration) and population-scale (e.g., mortality) rates. Each agent also carries state variables for *length* (*L*; mm), *age* (days), *gut fullness* (*GF;* proportion; 0–1), *energy reserves* (J), *maturity* (boolean), *visual range* (*r;* m), *migration status* (category to define movement behavior), and *grid position* (longitude [degrees], latitude [degrees], depth [m]). Predation and fisheries mortality operate on agent-level biomass; bioenergetic state variables (*energy reserves, GF, L*) are tracked per individual and multiplied by *abundance* when scaling to population fluxes. This superindividual approach reduces memory and computational requirements while preserving agent-level heterogeneity in size, position, behavioral state, and reproductive status. Initial agents are initialized by sampling a lognormal size distribution and accumulating agent biomasses until a user-specified target biomass density (metric tons [mt] km^-2^) is reached across the model domain, ensuring that aggregate biomass matches field estimates at initialization.

Population-level patterns are emergent properties of the cumulative actions of all agents responding to their local conditions. The implicit objective of each agent is to maximize fitness by surviving and accumulating surplus energy (*Energy*_*surplus*_) for growth and reproduction. Stochasticity is incorporated into habitat selection, prey capture, and reproduction to reflect natural randomness.

### Simulation modules

The simulation proceeds in discrete timesteps governed by a series of modules (Fig 2). The timestep duration dt (minutes) is user-specified. Time of day is tracked, with daylight hours currently defined as t ∈ [360, 1080) minutes (06:00–18:00). Future iterations could expand the daylight hours timing to include seasonal and latitudinal trends aligning with the study location.

**Fig 2:**
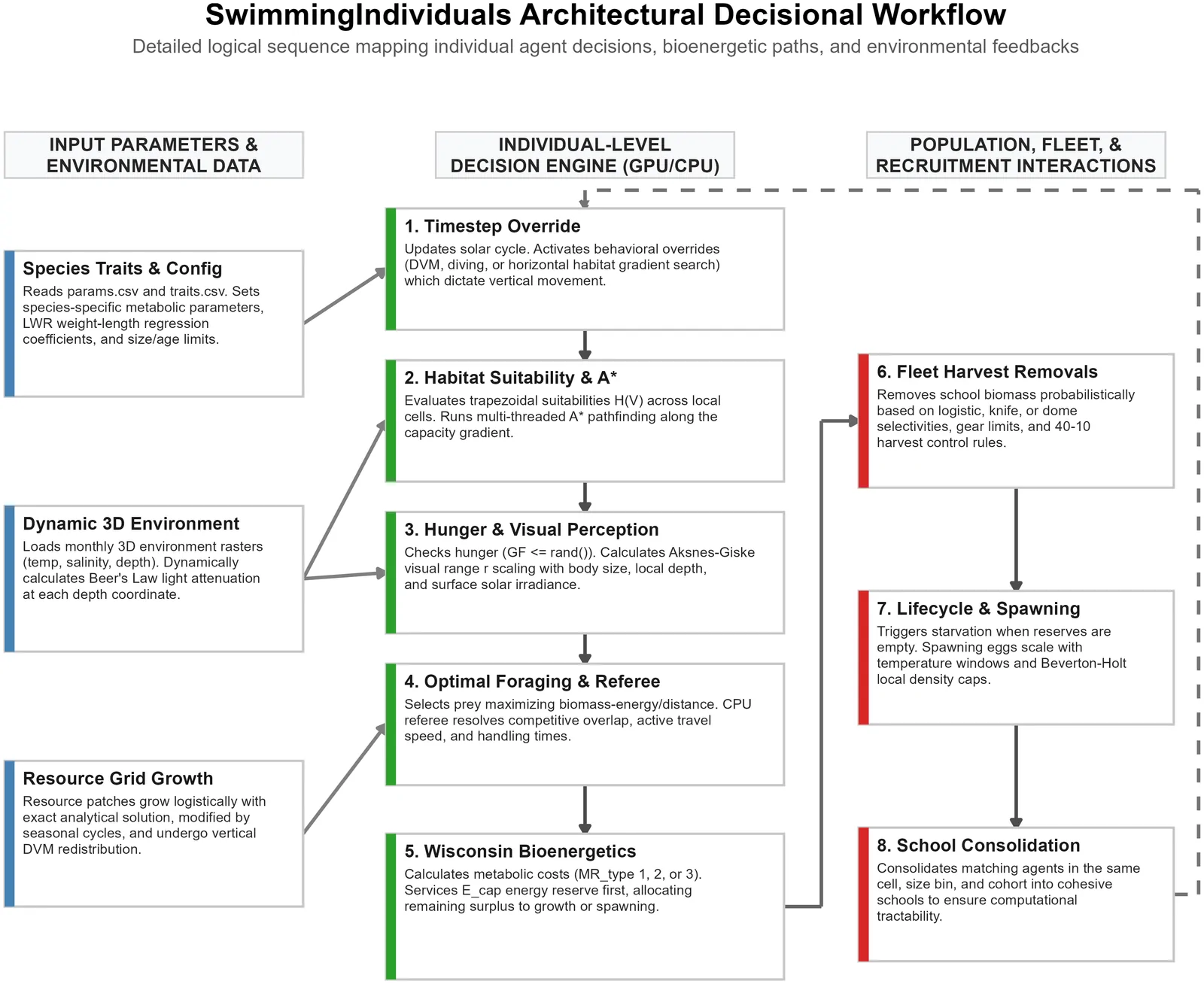
*SwimmingIndividuals* workflow, showing input environmental files, individual agent decision cycles, and emergent population, fleet, and recruitment interactions. In the workflow diagram, “A*” denotes the A* shortest-path algorithm used for habitat-seeking movement [12] and “Wisconsin bioenergetics” the standard Wisconsin energy-budget framework described below (Eq 4).

**Initialization:** The model creates focal species agents, resource patches, the gridded environment, and fishing fleets at simulation start. Agents are placed in the domain based on habitat capacity maps and their initial properties drawn from user-defined statistical distributions.

**Movement:** Agent movement is determined by its behavioral state. The default behavior moves agents toward higher-quality habitat via an A* pathfinding algorithm [12] constrained to cells with capacity > 0. This habitat-seeking movement can be overridden by specialized vertical movements for DVM or cetacean diving archetypes.

**Predation:** The predation submodel manages interactions through a three-step GPU-based sequence: record (each predator identifies the nearest suitable prey within its dynamically computed visual range), resolve (a CPU referee allocates available biomass among competing predators), and apply (a GPU kernel updates predator and prey state). An emergent functional response arises from gut fullness-dependent foraging probability.

**Bioenergetics:** The bioenergetics submodel computes a full Wisconsin-style energy budget [13,14] for each agent. It accounts for energy from consumption and costs from respiration, specific dynamic action, egestion, and excretion. Surplus energy is allocated to somatic growth and, for mature individuals, reproduction. Negative energy balance leads to starvation mortality. Three taxon-specific respiration sub-models currently exist in the model, which accommodate ectothermic fish, cetaceans, and deep-sea organisms.

**Fisheries:** The fisheries submodel applies fishing mortality to target and bycatch species via a GPU kernel. Biomass is removed probabilistically based on gear selectivity (logistic, knife-edge, or dome-shaped), quota constraints, season windows, and fleet-wide bag limits. The model tracks catch, effort, catch per unit effort (CPUE), and fishing mortality over space and time.

### Governing equations

The following sections provide the mathematical specification of each major submodel, implemented directly in the Julia programming language [15]. All parameters are defined where introduced.

1. **Visual Detection and Predator Perception**

An agent's visual range, *r* (m), is mechanistically determined at each timestep following Aksnes and Giske [8], as follows. Surface irradiance, *I*_*₀*_ (W m^-2^), follows a Gaussian diel curve over the daily cycle with peaks at solar noon, *I*_*0*_*(t) = I*_*peak*_ * exp(-(t_*h*_*-12)*^*2*^ */ (2σ*_*t*_^*2*^*)* with peak irradiance *I*_*peak*_ ~ 450 W m^-3^ at midday (*t*_*h*_ = hour of day), and a half-width *σ*_*t*_ = 4 h so that irradiance falls smoothly toward dawn and dusk. Light is attenuated with depth *z* (m) by Beer's Law (Eq 1):


$$\begin{array}{c} I_{z}=I_{\text{0}}e^{-k*z}, \end{array}$$ (1)

where the attenuation coefficient k = 0.32 – 0.016 * S, with *S* representing salinity (salinity) [16]. For a predator of body length, *L* (m), the maximum visual range is *r*_*max*_ = *30L*. The actual visual range is (Eq 2):


$$\begin{array}{c} =\sqrt{\frac{I_{z}*L_{prey}^{\text{2}}}{E_{s}*C^{\text{2}}}}, \end{array}$$ (2)

where *C* = 0.3 is inherent prey contrast, *L*_*prey*_ = *L* × *Prey*_*min_ratio*_ is the minimum prey body length considered (m), and *E*_*s*_ = *r*_*max*_*^2^* / (*P*_*image*_ × *C*) is the eye sensitivity, with *P*_*image*_ *= 0.75 × (L/0.01) × (L/4.0)* [17]. The resulting *r* is constrained to [0, *r*_*max*_]. Therefore, agents with larger bodies detect prey at greater distances, and detection is prey size-dependent; all agents have reduced visual range at depth and at night, producing emergent DVM and depth-structured predation without explicit programming.

2. **Predation Sequence**

At each timestep a GPU spatial index is built by assigning each agent a cell ID from its grid coordinates for efficient searching of nearby agents. This three-step predation sequence was formulated by pairing the logic of an optimal-foraging prey-selection rule [18] with a GPU-parallelized, atomic conflict-resolution scheme (i.e., multiple predators will not consume the same prey individuals), rather than adopting an existing algorithm.

**Step 1:** Each predator scans its horizontal neighborhood for prey within visual range *r* and within the accepted predator-prey size ratio [*Prey*_*min_ratio*_, *Prey*_*max_ratio*_] [19]. Rather than simply targeting the closest item, each predator applies an optimal-foraging rule and selects the most profitable prey, defined as the qualifying agent or resource grid cell that maximizes the energy-content-to-distance score profit = *(m  ×  ED) / (d^2^ + 1)*, where *m* is prey individual mass (g), *ED* is prey energy density (J g^−1^), and *d* is the predator–prey separation (km); the + 1 term bounds the reward for coincident prey. The profitability score therefore has units of J km^−2^. This profitability-weighted choice (a model design decision motivated by optimal foraging theory) is recorded as the predator's intent via atomic operations (“*atomic_cas!” in Julia*) that prevent race conditions among parallel GPU threads. Both resource patches and agents can be configured as suitable prey for a given predator and can therefore direct predator movement.

**Step 2:** A CPU referee collects all recorded intents and resolves conflicts when multiple predators target the same prey item. Available biomass is allocated among competing predators, and is stored in a *successful_ration* state variable for each predator that gets reset each timestep. The biomass any predator can claim in a step is capped by its remaining stomach space: the per-individual maximum stomach is allometric, *S*_*max*_ *= min(a*_*s*_ *× W*_*ind*_*^b*_*s*_*, W*_*ind*_*)*, and the school-level ingestion ceiling is *R*_*max*_ *= S*_*max*_ * abundance *(1 – GF_*t*_) * (dt / 1440), where *W*_*ind*_ is individual mass (g), *a*_*s*_ and *b*_*s*_ are species stomach-scaling coefficients, *GF*_*t*_ is current gut fullness [0–1], and *dt* is scaled from a daily capacity to the timestep. Both the per-individual *S*_*max*_ and *R*_*max*_ are in g, with *R*_*max*_ being the maximum biomass an agent can ingest per timestep. The allocated ration is therefore *min(available biomass, R*_*max*_*)*, so ingestion is jointly limited by prey availability and gut fullness rather than by a prescribed maximum-consumption parameter.

**Step 3:** A GPU kernel updates predator state (*Ration*_*energy*_*, Ration*_*biomass*_*, GF*) and prey state (*Biomass*_*school*_, *abundance*) based on resolved rations. Two physiological limits are imposed during application. First, the predator must reach its prey within the time remaining in the step: the travel time is *t*_*travel*_ *= d / (v × L)*, where *v* is swim speed in body lengths s ⁻ ¹, *L* is body length (m), *t*_*travel*_ is in seconds, and *d* is in meters; if *t*_*travel*_ exceeds the remaining time budget the prey is not captured, and otherwise the travelled time is deducted from the budget and added to active (swimming) time. Second, ingestion is bounded by handling time, *t*_*handle*_ (the time required to process one prey individual): the number of items a school can process is *n*_*handle*_ *= (t*_*remaining*_ */ t*_*handle*_*) × abundance*, the realized ration is *min(allocated ration, n*_*handle*_ *× m*_*prey*_ *× ED*_*prey*_*)*, and the time consumed in handling, *t*_*handle*_ *= (B*_*eaten*_ */ m*_*prey*_*) × t*_*h*_ */ abundance*, is then subtracted from the budget so that handling, searching, and travelling all compete for the same finite time. Successful consumption increments gut fullness by *GF*_*t*_ *= GF*_*t-1*_ *+ B*_*eaten*_ */ R*_*max*_, coupling each feeding event back to the hunger-dependent feed trigger.

Gut fullness, *GF*_*t*_, modulates foraging probability: a predator attempts to feed only if *GF*_*t*_ is less than a random number selected from a uniform distribution (0–1), creating a hunger-dependent feed trigger. Gut content evacuates at each timestep according to (Eq 3):


$$\begin{array}{c} {GF}_{t}={GF}_{t-\text{1}}*e^{\frac{dt}{\text{6}\text{0}}*ln\left(\text{1}-e_{prop}\right)}, \end{array}$$ (3)

where *e*_*prop*_ *= min(1.0, 0.053 × e*^0.073*T^*)* is a temperature (*T*; °C)-dependent evacuation rate [20] and *dt* is the timestep duration in minutes. After each predation event, predators that consumed prey and still have remaining time and incomplete guts continue foraging.

3. **Bioenergetics**

The energy budget follows the Wisconsin bioenergetics framework [13,14] (Eq 4):


$$\begin{array}{c} {Energy}_{Net}=C-R-(SDA+U+F), \end{array}$$ (4)

where *C* is energy consumed (J timestep^−1^ school^−1^), R represents respired energy (see below), and waste coefficients capture the energetic cost of individual behavior: Specific Dynamic Action (*SDA*) = 0.15 × *C*, Egestion (*U*) = 0.20 × *C*, and Excretion (*F*) = 0.08 × *C* [13]. These coefficients can be adjusted in the *energy.jl* source file. The respiration term, *R,* is computed by one of three taxon-specific sub-models selected by the *MR*_*type*_ species trait:

**MR**_**type**_ **1: Standard Ectotherm (default recommended for teleost fishes)**

The standard metabolic rate (*SMR*) of an individual (J timestep^−1^) is (Eq 5):


$$\begin{array}{c} SMR={resp}_{a}*W^{{resp}_{b}}*e^{{resp}_{q}*T}*W*oxy*\frac{dt}{\text{1}\text{4}\text{4}\text{0}}, \end{array}$$ (5)

where *W* is individual body mass (g), *T* is temperature (°C), *resp*_*a*_ is a mass-specific metabolic normalization coefficient (mg O_2_ g^−1^ day^−1^), *resp*_*b*_ is the allometric exponent, *resp*_*q*_ is the temperature coefficient, *oxy* = 13.6 J mg O_2_^−1^ is the oxycaloric equivalent, and *dt* is converted from daily to per-timestep units [21,22]. Total individual respiration (Eq 6):


$$\begin{array}{c} R_{ind}=SMR*\left(\text{1}-{active}_{time}\right)+\left(SMR*{activity}_{mult}\right)*{active}_{time}, \end{array}$$ (6)

Where *R*_*ind*_ is the respiration rate (J timestep^−1^) of each individual, *active*_*time*_ is the fraction of the timestep spent actively swimming, and *activity*_*mult*_ is a species-specific activity multiplier. School-level respiration is *R* = *R*_*ind*_ × *abundance*.

To keep mass-specific respiration physically bound at very small body sizes, the standard metabolic rate is capped at 2.5 mg O2 g^−1^ day^−1^. The implementation also auto-detects whether the supplied allometric exponent *resp*_*b*_ follows a mass-specific (negative) or whole-organism (positive) convention, so that published coefficients of either form can be used without double-scaling by mass; this is a model development decision intended to make the engine robust to the bioenergetics literature it is parameterized from.

**MR**_**type**_ **2: Endotherm (cetaceans)**

Field metabolic rate (*FMR*; J timestep^−1^) for cetaceans uses allometric scaling (Eqs 7 and 8):


$$\begin{array}{c} {FMR}_{min}=\text{3}\text{5}\text{0}*{\frac{W}{\text{1}\text{0}\text{0}\text{0}}}^{\text{0}.\text{7}\text{5}}*\text{4}\text{1}\text{8}\text{4}*\frac{dt}{\text{1}\text{4}\text{4}\text{0}}; \end{array}$$ (7)

$$\begin{array}{c} {FMR}_{max}=\text{4}\text{2}\text{0}*{\frac{W}{\text{1}\text{0}\text{0}\text{0}}}^{\text{0}.\text{7}\text{5}}*\text{4}\text{1}\text{8}\text{4}*\frac{dt}{\text{1}\text{4}\text{4}\text{0}}, \end{array}$$ (8)

where *W* is in grams and scaling coefficients (350–420 kcal kg day^−1^) are drawn from empirical cetacean bioenergetics literature [23]. Total individual cost is (Eq 9):


$$\begin{array}{c} R_{ind}=rmr\left(\text{1}-{active}_{time}\right)+rmr*{active}_{time}*{activity}_{mult}, \end{array}$$ (9)

where *rmr*, the resting metabolic rate is scaled between *FMR*_*min*_ and *FMR*_*max*_ by the fraction of the timestep an agent spends actively swimming (*active*_*time*_). The resting (*rmr[1-active*_*time*_*]*) and active components (rmr*active_*time*_) are then summed after the active fraction is inflated by *activity*_*mult*_. Because *rmr* increases with *active*_*time*_, total respiration scales more steeply than linearly with the active fraction, reflecting the elevated cost of sustained activity in endotherms.

**MR**_**type**_ **3: Deep-sea Organism**

For species permanently living in the deep sea, *SwimmingIndividuals* offers a simple, depth-dependent parametrization for respiration: metabolic rate declines with depth following empirical log-linear relationships [24] (Eq 10):


$$\begin{array}{c} n(R)=a_{\text{0}}+b_{\text{1}}*ln(W)-b_{\text{2}}\frac{\text{1}\text{0}\text{0}\text{0}}{\left(\text{2}\text{7}\text{3}.\text{1}\text{5}+T\right)}-b_{\text{3}}*ln(z), \end{array}$$ (10)

where *T* is temperature in Kelvin, and the intercept *a*_*0*_ and slopes *b*_*1*_, *b*_*2*_ vary by a user-input *taxa_code*: (1) *fish: a*_*0*_ *= 19.491, b*_*1*_ *= 0.885, b*_*2*_ *= 5.770, b*_*3*_ *= 0.261*; (2) *crustacean: a*_*0*_ *= 28.326, b*_*1*_ *= 0.779, b*_*2*_ *= 7.903, b*_*3*_ *= 0.365*; (3) *cephalopod: a*_*0*_ *= 18.775, b*_*1*_ *= 0.766, b*_*2*_ *= 5.265, b*_*3*_ *= 0.113* [24]. Oxygen consumption is converted to energy using *oxy* = 13.6 J mg O_2_^−1^, and individual respiration is computed as *R*_*ind*_ = *R* × *W* × (*dt*/60) scaled by *active*_*time*_ and *activity*_*mult*_.

4. **Surplus Energy Allocation**

Net energy surplus is computed at the school level and then partitioned between somatic growth and reproduction. For immature agents (*mature* = 0), all surplus energy goes to somatic growth. For mature agents, energy is partitioned based on relative body size:

Before any surplus is available for growth or reproduction, net energy is first stored as a physiological energy reserve (an internal buffer that is drawn down during deficits and refilled first during surpluses). The reserve capacity is set to the larger of a small floor (*E*_*min*_; default = 2.5 J ind^−1^) and 20% of the school’s body-energy content:


$$\begin{array}{c} E_{cap}=max\left(E_{min},\text{0}.\text{2}\text{0}*W_{ind}*N*ED\right); \end{array}$$ (11)

$$\begin{array}{c} {Energy}_{surplus}=max\left(\text{0},E_{net}-\left(E_{cap}-E\right)\right), \end{array}$$ (12)

where *N* is school abundance, a constant (2.5) in Eq. 11 represents a minimum reserve floor (*E*_*min*_) per individual, *W*_*ind*_ individual mass (g), and *ED* energy density (J g^−1^). Under a deficit (*E*_net_ < 0) the shortfall is drawn from the reserve *E*; under a surplus the reserve is refilled to *E*_*cap*_ first, and only the remainder is treated as surplus available for somatic growth and reproduction, which is then partitioned as follows (Eq 13–15):


$$\begin{array}{c} {repro}_{prop}=({\frac{L}{L_{max}})}^{\text{1}.\text{5}}; \end{array}$$ (13)

$$\begin{array}{c} {Energy}_{repro}={Energy}_{surplus}*(\text{1}-\text{0}.\text{1})*{repro}_{prop}; \end{array}$$ (14)

$$\begin{array}{c} {Energy}_{growth}={Energy}_{surplus}-{Energy}_{repro}, \end{array}$$ (15)

where *repro*_*prop*_ (Eq 13) is the size-dependent reproductive allocation fraction, which rises as *L* approaches *L*_*max*_, so that larger mature agents devote proportionally more surplus to reproduction; *Energy*_*surplus*_ (Eq 12) is the net surplus that is then partitioned into its reproductive share *Energy*_*repro*_ (Eq 14) and somatic share *Energy*_*growth*_ (Eq 15).

Growth energy is first scaled by a size-dependent somatic synthesis efficiency, *ε*_*som*_, that declines as the agent approaches its maximum size, and the resulting tissue energy is converted to individual biomass and added to mass (Eq 16), individual mass is updated, and new length is back-calculated from the length-weight regression parameters: *L = 10 × (W*_*ind*_ */ LWR*_*a*_*)^(1/LWR*_*b*_*)*. Maturation is triggered when the agent length is greater than or equal to the length-at-maturity (*L*_*mat*_; mm). Starvation mortality occurs when the agent's cumulative energy reserve is exhausted, i.e., when its reserve falls below zero (Eqs 11 and 12). In the current model version senescence is implemented as a strict maximum-age cutoff (Eq 17) rather than the exponential probabilistic hazard used during earlier development: an agent is removed once its age reaches the species-specific maximum age *A*_*max*_ (yr). This design decision was made to eliminate the gradual probabilistic attrition of long-lived cohorts that distorted the emergent age structure under the previous formulation, and to make the maximum lifespan an explicit, literature-constrained trait.


$$\begin{array}{c} \varepsilon_{som}=min\left(max\left(\text{0}.\text{4}\text{0}*\left(\text{1}-\text{0}.\text{5}*\frac{L}{L_{max}}\right),\text{0}.\text{1}\text{5}\right),\text{0}.\text{4}\text{5}\right);{Growth}_{biomass}=\frac{\varepsilon_{som}*E_{growth}}{N*ED}; \end{array}$$ (16)

$$\begin{array}{c} S_{sen}=\text{0}ifage\geq A_{max};S_{sen}=\text{1}otherwise, \end{array}$$ (17)

For mature agents, the reproductive share of the surplus (Eqs 13–15) is likewise scaled by a gonad synthesis efficiency of 0.45 before it is accumulated as the spawning reserve *repro*_energy_ that subsequently drives reproduction (Section 9, Eq 28). The somatic (Eq 16) and gonad efficiencies make the conversion of ingested energy into tissue explicit, so that growth and fecundity reflect biochemical synthesis costs rather than assuming lossless allocation.

5. **Habitat Quality and Habitat-Seeking Movement**

Habitat suitability for each species is defined by a piecewise linear trapezoidal function *H(V)* for each environmental variable *V* (Eq 18), governed by four user-supplied bounds per species per variable: specifying ranges of preferred and optimal habitat suitability, with *Pref*_*min*_ < *Opt*_*min*_ < *Opt*_*max*_ < *Pref*_*max*_


$$\begin{array}{c} H(V)=\text{0};ifV\leq {Pref}_{min}orV\geq {Pref}_{max}; \end{array}$$ (18a)

$$\begin{array}{c} H(V)=\frac{\left(V-{Pref}_{min}\right)}{\left({Opt}_{min}-{Pref}_{min}\right)};if{Pref}_{min}<V<{Opt}_{min}; \end{array}$$ (18b)

$$\begin{array}{c} H(V)=\text{1};if{Opt}_{min}\leq V\leq {Opt}_{max}; \end{array}$$ (18c)

$$\begin{array}{c} H(V)=\frac{\left({Pref}_{max}-V\right)}{\left({Pref}_{max}-{Opt}_{max}\right)};if{Opt}_{max}<V<{Pref}_{max}, \end{array}$$ (18d)

The composite habitat capacity score for each grid cell is the product of per-variable suitabilities across all preference rows for that species. Cells identified as land by a static bathymetry layer are assigned zero capacity (land/ocean polarity is detected automatically from the deep-domain reference cell), which, because both initial placement and movement are driven by habitat capacity, confines agents to water. These scores are precomputed for all months and stored as a 4D capacity array (longitude, latitude, month, species). As the simulation progresses, the capacities are indexed by month.

At each timestep, agents with remaining time budget attempt habitat-seeking movement. The A* algorithm finds the shortest path (using Euclidean distance as a heuristic) [12] from the agent's current cell to a goal cell selected probabilistically, weighted by habitat capacity, from the local neighborhood reachable within the agent's per-step displacement. Diagonal moves are allowed (time cost = 1.414 vs. 1.0 for cardinal directions). The agent advances along this path by a distance budget determined by its swim speed (body lengths s^−1^ × body length × remaining time). Paths are memorized within each timestep using a thread-safe dictionary to avoid redundant A* calls. Agents in zero-capacity cells, which can occur during monthly environment shifts, are first relocated to the nearest suitable habitat via breadth-first search before A* path planning. To prevent unrealistic basin-scale relocation within a single long timestep, net horizontal displacement is bounded by a per-step cap derived from a user-supplied maximum daily net displacement (*Move_Max_km_day*; default 60 km day^−1^), and the goal cell is drawn from cells reachable within that cap. Agents therefore track the seasonally shifting habitat optimum by climbing the local capacity gradient rather than redistributing across the basin each step [25].

6. **Diel Vertical Migration and Behavioral Archetypes**

Four behavioral archetypes govern vertical movement: nonmigratory (habitat-seeking only), strong diel vertical migration (i.e., each individual migrates every night), weak diel vertical migration (i.e., each individual migrates only when it needs to eat) [26], and diving. For DVM archetypes, target depths are drawn from a tri-modal Gaussian mixture distribution (Eq 19) at each diel transition:


$$\begin{array}{c} p_{z}=\lambda_{\text{1}}*N\left(\mu_{\text{1}},\sigma_{\text{1}}\right)+\lambda_{\text{2}}*N\left(\mu_{\text{2}},\sigma_{\text{2}}\right)+\lambda_{\text{3}}*N\left(\mu_{\text{3}},\sigma_{\text{3}}\right), \end{array}$$ (19)

with separate parameter sets for day and night distributions. Agents currently migrate toward their target depth at a fixed vertical swim speed of 4 m min^−1^ [27], but this constant can be made more dynamic by modifying the *behavior.jl* file. Weak migrants additionally require a gut fullness check (*GF < rand(Uniform[0,1])*) before ascending, so satiated individuals forgo that night’s ascent. Daytime descent begins at *t* = 360 min (06:00) and nigh*t*time ascent at *t* = 1080 min (18:00). A *mig_status* state variable (0 = surface, 1 = ascending, 2 = descending, −1 = at depth) prevents simultaneous horizontal habitat-seeking during migration.

For diving agents [28], dives are triggered probabilistically when *GF < 0.5 × rand(Uniform[0,1])*. Ascent is triggered upon reaching target depth; the agent returns to *z* ≤ 5m to complete the dive cycle. We note that both DVM and diving are currently implemented as archetype-driven overrides rather than emergent consequences of predator avoidance, representing a potential direction for future model development.

7. **Fisheries**

Each fishery is defined by a Fishery struct (name, quota, season, area, slot limits, bag limits, selectivities). Three gear selectivity types are implemented that provide a length-based selectivity value, *S(L)*, for each fishery-species interaction. Option 1 incorporates a logistic function where selectivity increases with increasing agent size, driven by a user-provided *slope* and the midpoint parameter *L*_*50*_ (Eq. 20). Option 2 uses a threshold length (*L*_*Knife*_), which simulates an extreme shift in selectivity (Eq. 21). A dome-shaped selectivity function also exists for fisheries that have both lower and upper length selectivity thresholds (Eq. 22).


$$\begin{array}{c} S(L)=\frac{\text{1}}{\left(\text{1}+e^{-slope*(L-L_{\text{5}\text{0}})}\right)}; \end{array}$$ (20)

$$\begin{array}{c} S(L)=\text{1};ifL\geq L_{Knife};else:S(L)=\text{0}; \end{array}$$ (21)

$$\begin{array}{c} S(L)=\frac{\text{1}}{\left(\text{1}+e^{-{slope}_{\text{1}}*(L-L_{\text{5}\text{0}\_\text{1}})}\right)}*\left(\text{1}-\frac{\text{1}}{\left(\text{1}+e^{-{slope}_{\text{2}}*\left(L-L_{{\text{5}\text{0}}_{\text{2}}}\right)}\right)}\right), \end{array}$$ (22)

At each timestep, for each active fishery, a GPU kernel evaluates each living agent. Agents within the fishery's geographic area and slot size limits are subjected to a probabilistic removal based on the length-based selectivity value (0–1) and the selection of a uniform random value (also 0–1). Individuals within the agent are harvested if the random value is less than the selectivity metric. Atomically accessed arrays track remaining quota (metric tons) and fleet-wide bag limits (the product of vessel bag limits and the number of vessels). When annual quota is exhausted, no further removals occur until the next year. Catch statistics (biomass, individuals, mean length, CPUE) are accumulated per fleet per timestep. Quotas reset annually at the first timestep of each calendar year. Each fleet’s annual quota may be fixed or governed by a biomass-linked harvest control rule [29,30]: in the latter case the quota is recomputed each year as the product of a baseline quota and the ratio of current to reference target-species biomass, so the allowable catch tracks stock status (rising when the stock is healthier and falling when it is depleted); alternatively a constant exploitation rate applied to current biomass, an optional biomass ramp that reduces the quota toward zero below a limit biomass, and lower and upper quota caps may be specified.

When a fleet is placed under a biomass-linked harvest control rule, its annual quota is recomputed each year from the current target-species biomass *B* (mt). Either a constant exploitation rate *h* is applied to current biomass, or the baseline quota is scaled by the ratio of current to reference biomass (Eq 23a):


$$\begin{array}{c} Q=h*BorQ=Q_{base}*\frac{B}{B_{ref}}\text{.} \end{array}$$ (23a)

The optional biomass ramp then scales this quota linearly to zero between a threshold biomass *B*_*thr*_ and a limit biomass *B*_*lim*_, and lower and upper caps bound the result (Eq. 23b):


$$\begin{array}{c} \varphi =clamp\left(\frac{B-B_{lim}}{B_{thr}-B_{lim}},\text{0},\text{1}\right);Q=clamp\left(\varphi *Q,Q_{min},Q_{max}\right)\text{,} \end{array}$$ (23b)

so that the allowable catch rises when the stock is healthy and is throttled toward zero as biomass approaches the limit reference point. Julia utilizes a *clamp()* function to constrain a calculated value between a particular range (e.g., 0–1 in Eq. 23b). With the default columns these rules reduce exactly to the fixed-quota behavior, so the dynamic control rule is opt-in per fleet [29,30].

8. **Natural Mortality and Resource Dynamics**

Background natural mortality for focal species is applied per agent per timestep as continuous exponential decay (Eq 24):


$$\begin{array}{c} S_{dt}=e^{\frac{-M*dt}{\text{5}\text{2}\text{5}\text{6}\text{0}\text{0}}}\text{,} \end{array}$$ (24)

where *M* is the annual instantaneous natural mortality rate (yr^−1^) and 525,600 is minutes per year, so that *M* is the per-minute rate corresponding to the temporal scale of *S*_*dt*_; the resource-mortality equation (Eq 27) uses the identical per-minute convention. Abundance, biomass, and energy are all scaled by *S*_*dt*_ per agent. Resource grid biomass follows logistic growth with the exact analytical solution:


$$\begin{array}{c} B(t)=\frac{K}{\frac{\left(\text{1}+\left(\left(K-B_{t-\text{1}}\right)\right)\right)}{B_{t-\text{1}}}*e^{-r_{adj}}}\text{.} \end{array}$$ (25)

Mid-trophic resource groups are not passive: in addition to growing logistically (Eq 25) [31], each resource group forages on co-located agents and other resources through a Holling Type II functional response [32]. For a predatory resource the per-unit biomass consumption rate in a cell is given by Eq 26,


$$\begin{array}{c} C_{R}=\frac{a*\rho }{\text{1}+a*t_{h}*\rho }*\frac{dt}{\text{1}\text{4}\text{4}\text{0}}*{ED}_{avg}\text{,} \end{array}$$ (26)

where *a* is the clearance (searching) rate set by the group’s daily ration, *ρ* is the local prey energy density (J m^-3^), *t*_h_ is handling time (days), and *ED*_avg_ is the biomass-weighted mean energy density of available prey. Realized consumption is capped at the group’s maximum daily ration, and a fixed 15% of the consumed prey biomass is returned to the resource grid as trophic growth, *ΔB* = 0.15 × (consumed biomass), closing a simple mid-trophic energy loop without resolving those organisms as individual agents.

where *K* is the cell-specific carrying capacity (g; default = 2x the initial cell biomass) and *r*_*adj*_ = *r*_*annual*_ × *seasonal_multiplier / (minutes_per_year / dt*) is the per-timestep growth rate after applying monthly seasonal multipliers from a user-supplied table. When a relative primary production (*PP*) layer is provided in the environmental configuration, the cell carrying capacity *K* is additionally scaled by a normalized *PP* multiplier, *PP*_*t*_*/mean(PP)* clamped to [0.2, 5]; productivity is applied to the standing-stock capacity *K*, meaning that changing *PP* conditions affect the maximum standing stock the grid cell allows. Background resource mortality is applied after growth:


$$\begin{array}{c} B_{t}=B_{t-\text{1}}*e^{\frac{-M_{r}*dt}{\text{5}\text{2}\text{5}\text{6}\text{0}\text{0}}}\text{,} \end{array}$$ (27)

where *M*_*r*_ is the annual instantaneous mortality rate that is not already implemented in the model (e.g., predation mortality from non-modeled species; unaccounted fishing mortality). Resource grids are redistributed monthly according to updated habitat capacity maps, and diel vertical redistribution occurs at each day/night transition using the same Gaussian mixture profiles as agent DVM.

Source-specific instantaneous mortality rates are recorded for focal agents. Over each output interval the model accumulates the biomass removed by predation (*P*; both resource-on-agent and agent-on-agent consumption), starvation (*S*), fishing (*F*), and other causes (*O*; e.g., senescence). Each accumulated kill biomass is converted to an instantaneous rate against the standing biomass-by-size at the start of the interval, *B*_*ref*_, to convert timestep-specific mortality rates into relatable, annualized metrics. Rates are written both spatially explicitly (per grid cell, species, and size bin; *F* additionally per fleet) and as a spatially integrated per-interval time series (one value per output for each source).

9. **Reproduction**

Mature agents that accumulate *Energy*_*repro*_ > 0 during the spawning season trigger the reproduction function. The number of eggs produced by *parent*_*i*_ is:


$$\begin{array}{c} N_{eggs}=\frac{\left({Energy}_{repro}*{season}_{mult}\right)}{\left({Energy}_{egg}+\varepsilon \right)}*{sex}_{ratio}*{survival}_{hatch}\text{,} \end{array}$$ (28)

where *Energy*_*egg*_ = 2.15 × *egg*_*volume*_^0.77^ (J egg^−1^) with *egg*_*volume*_ = 0.15 × *W*_*ind*_^0.14^; *sex*_*ratio*_ is the fraction of spawning individuals that are female (assuming agents are comprised of both male and female individuals), *season*_*mult*_ is a user-provided multiplier [0–1] that allows for monthly differences in spawning season and *survival*_*hatch*_ is the fraction of eggs that survive to recruitment. Each producing parent generates one new agent with the abundance equal to the number of eggs rounded down to the nearest integer and placed at the parent's location with larval biomass and size. This egg production formula corresponds to the creation of all offspring for all archetypes, including cetaceans and other species that give birth to live offspring. In these cases, the *larval_size* parameter should be large (default = average size at birth) and *survival*_*hatch*_ parameter should be small to limit egg production to realistic levels.

Egg production (Eq. 28) is converted into realized recruits by three multiplicative survival and process terms that translate environmental conditions and stock density into recruitment, each reducing to unity under its default so the deterministic case is recovered. First, early-life survival follows a Gaussian thermal-performance window evaluated at the egg’s ambient temperature *T*, *exp(−0.5((T − T*_*opt*_*)/T*_*sd*_*)²)*, with optimum (*T*_*opt*_) and width (*T*_*sd*_), capturing temperature control of spawning and larval survival [33,34]. Second, survival is reduced by local conspecific spawner biomass (parent biomass + other spawners in the grid cell; *SSB*_*local*_) as *1/(1 + β·SSB*_*local*_*)*; because egg production scales approximately linearly with local spawner energy while per-egg survival declines with local density, the realized recruits-SSB relationship saturates, so a Beverton–Holt compensatory relationship [35] emerges from local crowding rather than being imposed at the stock level [36]. Third, a single cohort-level lognormal process-error deviation, *exp(σ*_*R*_*·θ − σ*_*R*_*²/2)* with *θ ~ N(0,1)*, introduces interannual recruitment variability following standard stock-assessment practice [37]. The compensation parameter *β* is specified as *1/dd*_*halfsat*_ (the local spawner biomass at which early survival halves), and *T*_*opt*_*, T*_*sd*_*, σ*_*R*_*, and dd*_*halfsat*_ are supplied per species in an optional *recruitment_env.csv* file.

School consolidation runs each timestep, grouping agents sharing the same grid cell, size bin, generation, and birth cohort. Smaller schools are merged into larger anchors via abundance-weighted averaging of intensive properties (length, gut fullness, spatial coordinates) and summation of extensive properties (biomass, energy, abundance), provided the merged school does not exceed a user-provided *school*_*size*_.

### Model inputs and configuration

*SwimmingIndividuals* is configured through a set of user-provided CSV, XML, and ESRI ASCII raster input files referenced by a master manifest (*files.csv*). The master manifest (*files.csv*) stores file paths to all other input files, allowing users to switch between scenarios by modifying a single pointer file rather than editing source code. The simulation parameters file (*params.csv*) specifies high-level run settings including the number of focal and resource species, timestep duration (*model_dt*, in minutes), total timesteps (*nts*), output frequency, and the spin-up period during which mortality is suppressed to allow agents to reach energetic equilibrium. Species traits are provided separately for focal agents (*traits.csv*) and resource groups (*resource_traits.csv*). Each row represents a species. Environmental preference bounds for the habitat suitability function are specified in *envi_pref.csv*, with one row per species per environmental variable. The environmental driver data are supplied as monthly ESRI ASCII (.asc) rasters, one file per variable per month, catalogued by an XML configuration (*environment.xml*) that defines, for each variable, its role (habitat driver, relative primary production, or static field such as bathymetry), the spanned time period, and a filename pattern; rasters are resampled onto the model grid by nearest geographic cell center. Required variables include temperature (°C), salinity (PSU), and bathymetry (m) fields, the last masking land cells (capacity set to zero) so agents are confined to water. An optional recruitment_env.csv supplies per-species recruitment parameters (*T*_*opt*_, *T*_*sd*_, *σ*_*R*_, *dd*_*halfsat*_), and the simulation start date is set in params.csv (*start_year, start_month, start_day*). Fishery regulations are loaded from *fisheries.csv*, with one row per fleet, specifying the fleet name, target and bycatch species codes, annual quota (metric tons), season bounds (days of year), geographic area bounds (latitude and longitude), slot size limits (mm), vessel count (n vessels), daily bag limits (ind. vessel^−1^ day^−1^), and gear selectivity parameters. The full list of model input parameters is in the Supplemental Appendix 1. All input files for the three case studies presented here are included in the public GitHub repository (https://github.com/mattwoodstock/SwimmingIndividuals) directory of each scenario folder, and the online user guide documents every parameter with units, acceptable ranges, and default values.

### Comparison to existing ABM and IBM frameworks

Several established ecosystem modeling frameworks address related problems in marine ecosystem modeling. Ecospace [31] operates on aggregated biomass pools organized by functional group, meaning that individual heterogeneity and emergent behavioral dynamics cannot be captured. Atlantis [38] similarly structures the ecosystem using age-structured functional groups rather than discrete individuals, coupling end-to-end biogeochemical and fisheries dynamics at the cost of individual-level resolution. OSMOSE [25] advances beyond these pool-based frameworks by tracking discrete schools as superindividuals that move spatially and interact through size-based predation; however, its predation sub-model relies on empirical stochastic accessibility coefficients rather than mechanistic visual detection, it lacks an explicit energy-budget bioenergetics framework and a three-dimensional depth structure. NetLogo-based IBMs [39] are similarly constrained with regards to the processes that are being modeled. SEAPODYM [33] simulates individual tuna movements but focuses on a single species at a time and does not implement a full multi-species predation and bioenergetics framework. Ichthyop [40] simulates larval dispersal as individual particles but lacks bioenergetics, predation interactions, and adult life history. Although these frameworks have demonstrated capacity within marine conservation and fisheries applications, *SwimmingIndividuals* offers a distinct solution to integrating physics and behavioral components within a bioenergetics and ecosystem modeling context.

Relative to these frameworks, *SwimmingIndividuals* offers three specific technical advances. First, agent perception is governed by a physics-based visual detection model derived from light attenuation theory, such that a predator's detection radius scales dynamically with body size, depth, and ambient irradiance at each timestep, rather than using a fixed radius. Second, the bioenergetics engine currently accommodates three distinct metabolic architectures within a single framework simultaneously: standard ectothermic fish (mass- and temperature-dependent respiration), endothermic cetaceans (allometric field metabolic rates), and deep-sea organisms (depth- and log-linear temperature-dependent metabolism), allowing multi-taxon ecosystems to be simulated with taxon-appropriate physiology. Third, the hybrid CPU/GPU architecture, implemented in Julia using *KernelAbstractions.jl*, enables GPU-parallelized spatial indexing, predation kernel execution, and fisheries removal alongside CPU-threaded bioenergetics and pathfinding, enabling the modeling of large populations of superindividual agents. Together these advances allow *SwimmingIndividuals* to conduct in-silico experiments at ecosystem scales that are not currently achievable within any single existing ABM framework.

## Results

To demonstrate the capabilities of *SwimmingIndividuals*, we conducted three targeted simulations in the Gulf of America: a one-week DVM scenario with two vertically migrating species; a two-day Diver scenario with two deep-diving cetacean-like species; and a 30-year Mackerel scenario with King Mackerel (*Scomberomorus cavalla*) and nineteen resource groups. Based on the 3 simulations, we showcase the model's ability to reproduce complex ecological phenomena as emergent properties below.

### Emergent movement patterns

Simulation of individual agent paths yielded highly structured, emergent vertical and horizontal behaviors. Diel vertical migration (DVM) profiles (Fig 3A) for the strong migrant population demonstrated an abrupt, coordinated descent to depths between 300-600m at the onset of dawn, followed by a rapid ascent back to surface waters ~ 50m at dusk. The majority of the agents in the weak migrant species (only a proportion of the population migrates to shallower water each night) migrated, but a small proportion (< 5%) did not, ostensibly because of a full stomach. These patterns were driven by the hunger state of the agents, as well as a diel vertical migration archetype (weak or strong diel vertical migration). In a second, cetacean diving simulation, agents replicated the high-frequency foraging dives and regulatory breathing intervals typical of deep-diving endotherms (Fig 3B). The deep diver analogue executed rapid, steep descents to depths between 400-650m, maintaining a bottom foraging phase before ascending to the surface (~5m). These repeated diving cycles generated a highly bimodal travel distance profile, where 3D daily travel distances for individual diving agents ranged between 40–160km per day. This wide distribution arose directly from the literature-derived dive characteristics of each cetacean species and the swimming velocity of the animal. Future configurations of the DVM and diving archetypes may release their input-driven vertical movements to produce emergent processes, driven by the optical properties of the water column and prey availability.

**Fig 3:**
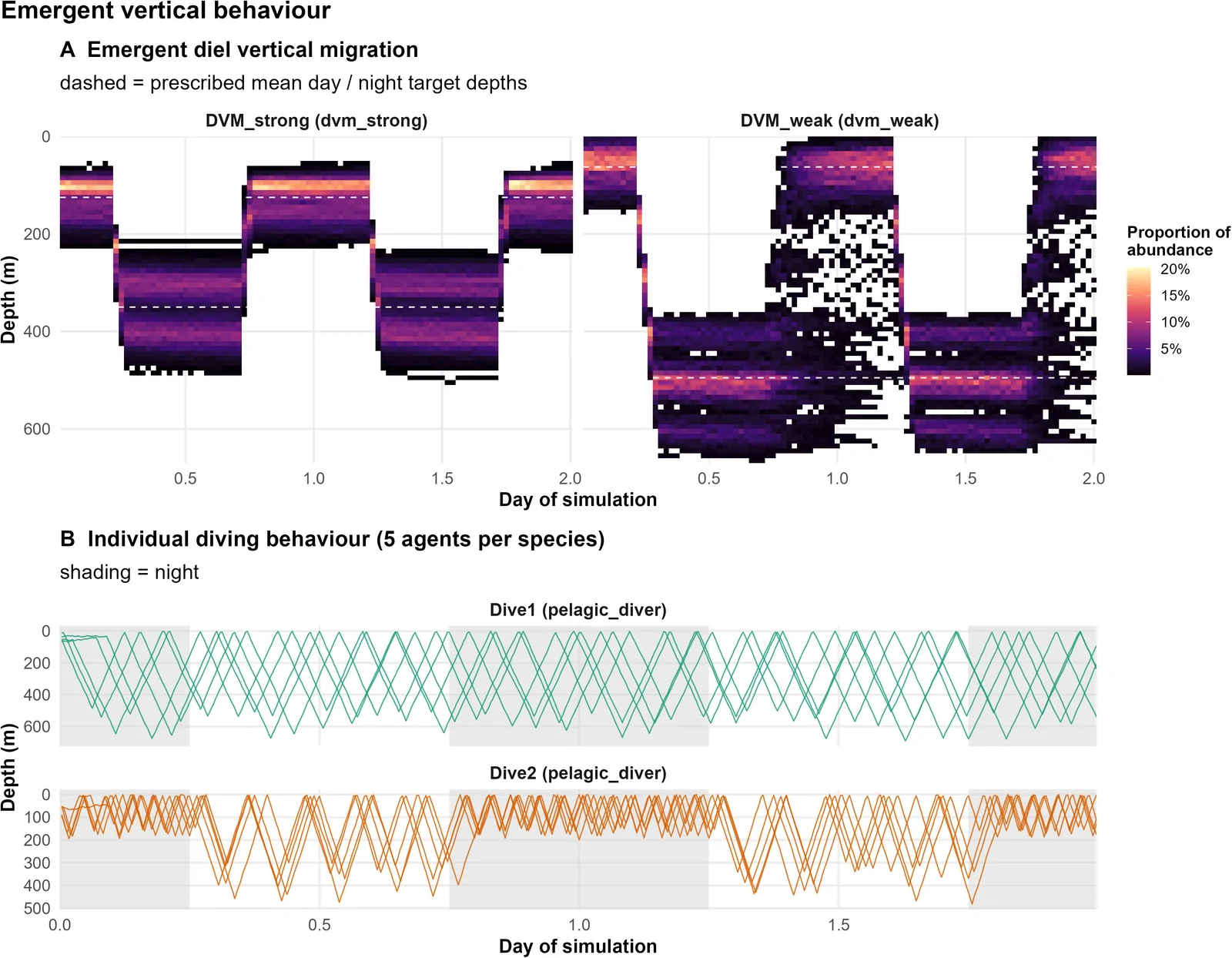
Emergent vertical movement patterns. **A)** Depth distribution profiles of strong and weak diel vertical migrant populations over a one-week period, with aggregation densities indicated by color gradients (darker blue represents higher density). **B)** High-resolution dive profiles of deep-diver (Dive1; sperm whale [*Physeter macrocephalus*] analogue) and diel-diver (Dive2; Rice's whale [*Balaenoptera ricei*] analogue) cetacean agents over a 50-hour window, showing individual-level dive cycles (5 individuals represented per species by distinct colors).

On a larger scale, seasonal migrations emerged organically from localized, step-by-step movement decisions (Fig 4). In January, under cooler winter temperatures, the simulated King Mackerel population concentrated in high-capacity waters along the southeastern coastal shelf, where the piecewise linear trapezoidal preference function scored the environmental fields as more preferrable. This follows literature that indicates that King Mackerel migrations are highly temperature and salinity-driven [41]. As the year progressed and water temperatures warmed, the spatial distribution of the habitat suitability index shifted northward and westward. By May, the mackerel agents had navigated along these seasonal gradients, relocating to the northern shelf. This large-scale redistribution emerged without any pre-programmed migration routes or coordinate targets; agents traversed along local suitability gradients during each horizontal movement step, demonstrating that basin-scale migrations can arise from fine-scale directed movements.

**Fig 4:**
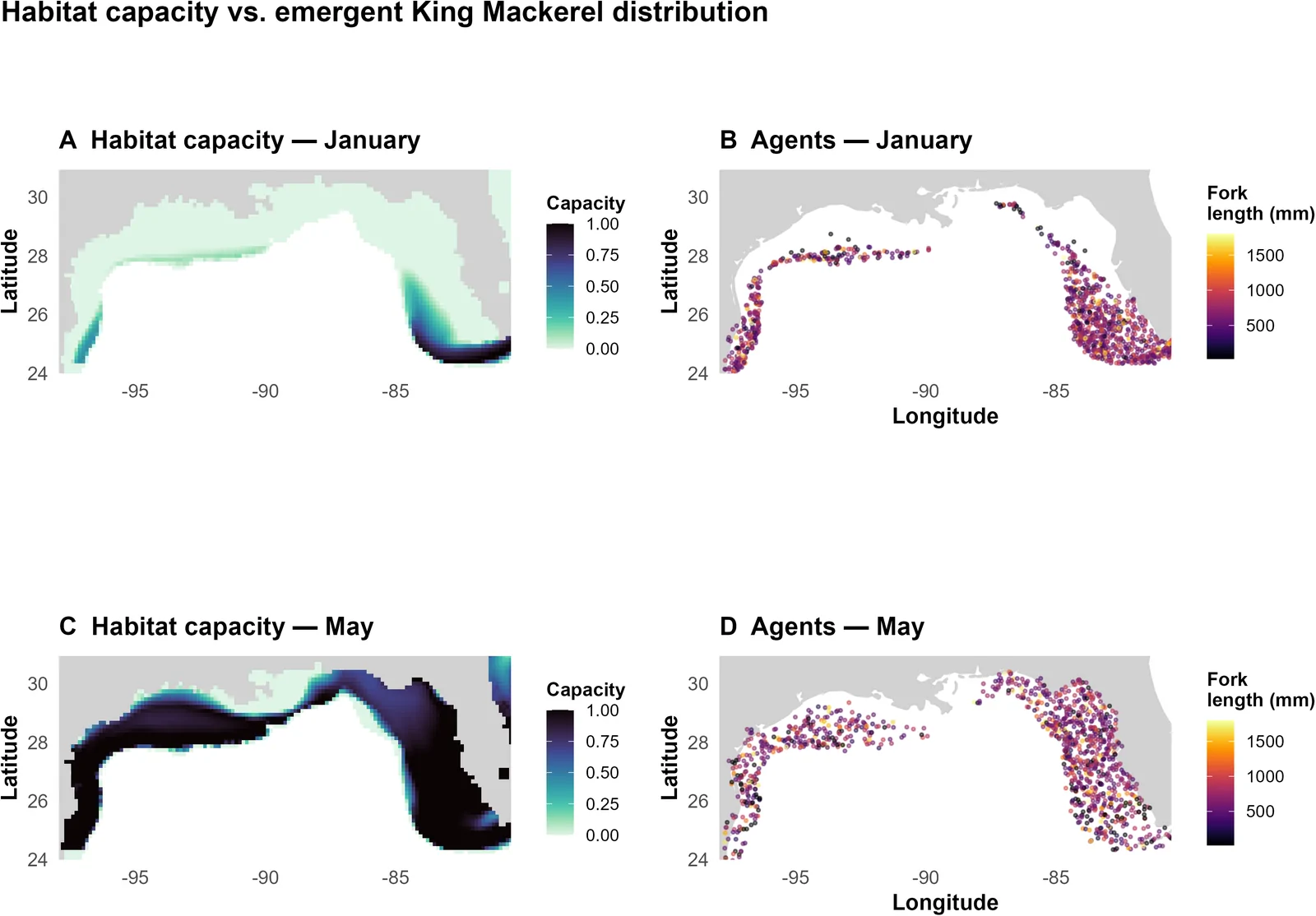
Emergent spatial distribution of King Mackerel agents compared with monthly habitat suitability maps. Panels A and C show the spatial distribution of the precomputed multi-variable habitat capacity score (0.0 to 1.0; see Section 4) for January and May, respectively. Panels B and D show the corresponding geographical coordinates of simulated superindividual agents, colored by individual fork length (mm), illustrating basin-scale migration along seasonally shifting gradients.

### Predator-prey and trophic dynamics

Trophic interactions resolved by the multi-species predation module produced a highly structured, self-organizing marine food web (Fig 5A). This food-web structure was an emergent property of the time- and stomach-limited foraging algorithm: because each predator can attempt only a bounded number of captures per timestep and stops feeding once its gut fills, per-capita intake saturates with prey density, producing a Type-II-like functional response at the population level rather than an imposed Holling curve. The calculated fractional trophic levels ranged from 2.0 for primary consumers to 4.8 for apex sharks and large pelagic teleosts. King Mackerel agents exhibited ontogenetic trophic separation across their ten tracked size classes (ten evenly distributed size classes): small juveniles (Bins 1–3; FL < 300mm) fed exclusively on pelagic invertebrates and primary resource groups, occupying a trophic level near 3.1, whereas large adults (Bins 8–10, FL > 1000mm) transitioned to consuming small tunas, Spanish Mackerel, and other mid-trophic fishes, elevating their fractional trophic level to 4.2-4.5. This ontogenetic diet shift (Fig 5B) emerged without manual dietary parameterizations or preference parameters; visual predation was restricted by size-ratio thresholds, meaning that as agents grew somatically, their visual detection range for larger prey expanded according to the Aksnes-Giske model, and the CPU referee allocated larger biomass portions to them based on their expanding stomach volumes. Predation mortality exhibited distinct predation “hotspots” along the continental shelf break of the Gulf (Fig 5C). The 60-year mean spatial predation rate on King Mackerel agents was heavily concentrated in waters between 50-400m, where high prey densities and favorable temperatures attracted both mackerel and larger apex predators. This spatial heterogeneity demonstrates that the risk of mortality is tightly linked to physical-biological overlaps that concentrate trophic interactions into localized regions.

**Fig 5:**
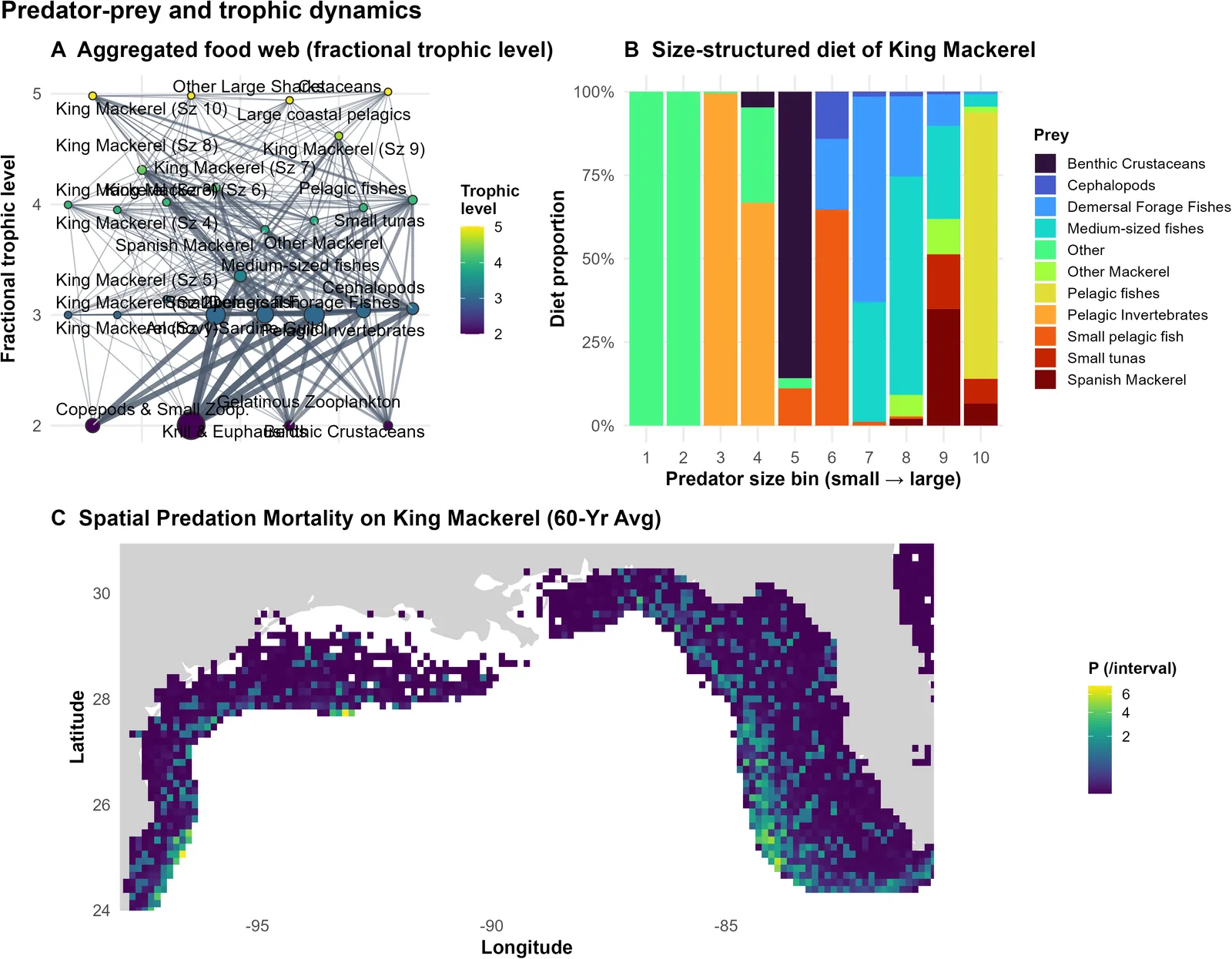
Emergent predator-prey and trophic structure from the 60-year multi-species simulation. **A)** Spatially aggregated food web network diagram, where node vertical coordinates represent calculated fractional trophic levels, node sizes scale with total biomass throughput, and line weights represent the magnitude of trophic transfer. **B)** Size-structured diet composition of King Mackerel across its ten life-history size bins (small to large), illustrating the emergent ontogenetic transition from planktivory to piscivory. **C)** Spatial distribution of mean predation mortality rates (in annualized instantaneous rates) on King Mackerel agents in the Gulf of Mexico, highlighting localized predator-prey overlap hotspots along the coastal shelf.

### Life history and bioenergetics

Physiological state variables tracked at the agent-based level demonstrated high consistency with empirical metabolic and somatic constraints (Fig 6). Individual reserve energy density (Fig 6A) remained highly stable across all size classes, clustering tightly around 1200J g^−1^ for mature King Mackerel individuals (FL > 600mm). Notably, the biphasic energy allocation for somatic and gonadal growth puts a cap on reserve energy density once an animal reaches maturity, as excess will be utilized for either maintenance, somatic growth, or egg production. Values below the maximum energy density indicate individuals must refill their energy reserves before growth or reproduction. Higher individual-level variance was observed among young juveniles (FL < 300mm), where rapid structural growth requirements and high mass-specific respiration rates periodically depleted energy reserves to near-starvation levels (< 500 J g^−1^) during winter resource declines, demonstrating the bioenergetic vulnerability of early life stages to local environmental fluctuations.

**Fig 6:**
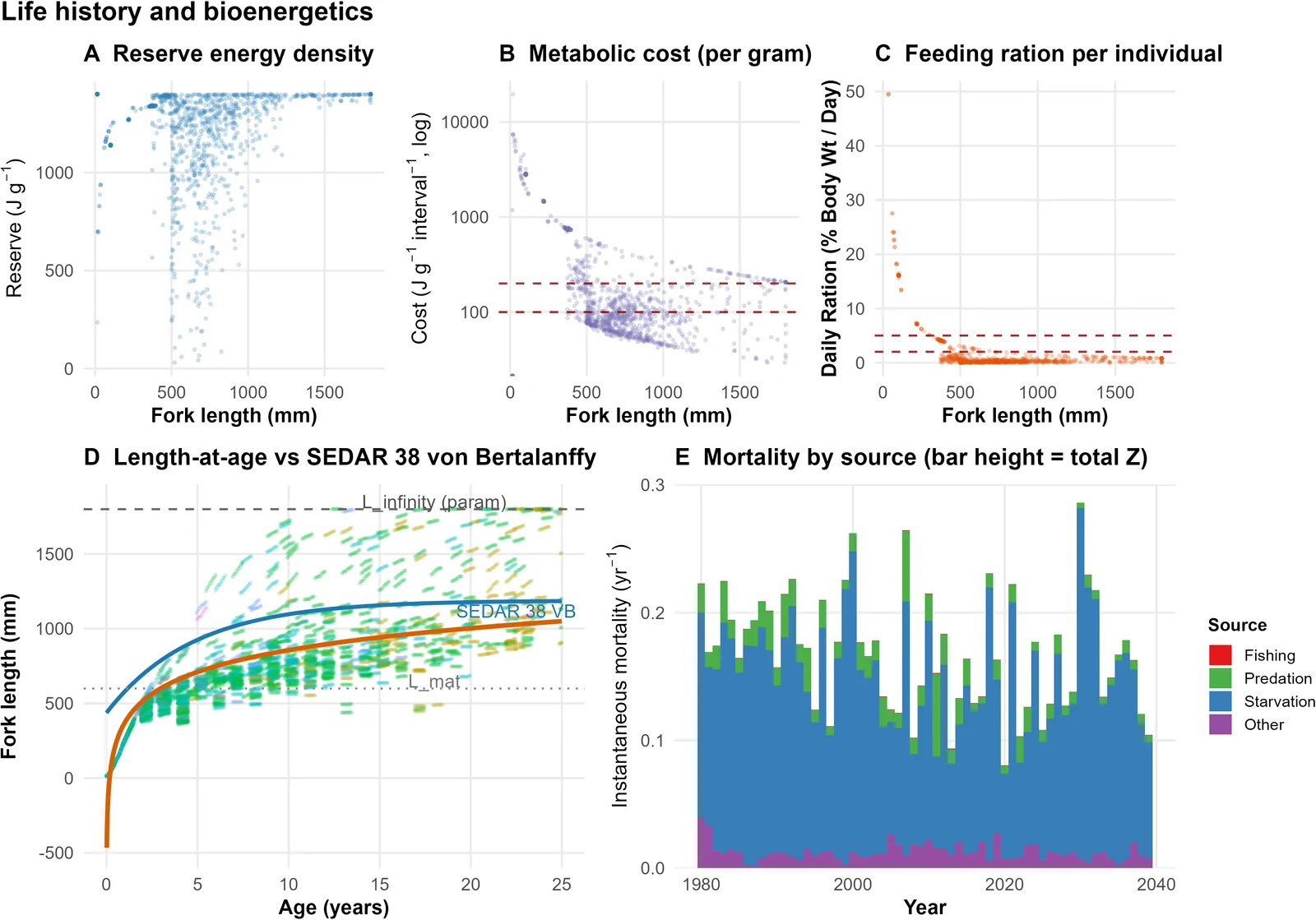
Individual and population-level bioenergetic and life-history outputs for King Mackerel. **A)** Reserve energy density (J g^−1^) across different individual lengths (mm). **B)** Mass-specific metabolic cost (J g^−1^ timestep^−1^) plotted on a log scale; dashed horizontal lines indicate the physiological standard metabolic rate boundaries (100-200 J g^−1^ timestep^−1^) for active pelagic scombroids from published literature [42,43]. **C)** Emergent daily feeding ration (% body weight per day) across individual lengths, with dashed lines indicating the adult scombroid foraging bounds (<% bodyweight consumed per day) from literature [44]. **D)** Somatic length-at-age growth curves across multiple cohorts, showing the logarithmic model fit, maximum body size (1800 mm), length at maturity (600m), and the latest stock assessment von Bertalanffy growth curve (blue line; [37]). **E)** Spatially integrated instantaneous mortality rates partitioned into fishing, starvation, predation, and other background sources over the full simulation.

Mass-specific metabolic costs (Fig 6B) exhibited a standard power-law decline with body size, settling at 100–200 J g^−1^ timestep^−1^ for adult scombroids (FL > 600mm). This energetic baseline converges directly with the resting and active standard metabolic rate (SMR) boundaries measured for pelagic scombroids in laboratory flumes by [42,43], validating that the model's SMR scaling and active swimming multipliers reproduce realistic thermodynamic costs for active marine swimmers. Daily foraging rations (Fig 6C) demonstrated a matching decline with size, with adult rations settling under a 5% of body weight per day average. This adult consumption rate is highly consistent with empirical stomach-content analyses of coastal migratory pelagics compiled by [44], confirming that the visual predation module can represent adult foraging rates. Conversely, small juveniles (FL < 200mm) exhibited highly elevated and variable daily rations (10%-30%), which were necessary to sustain their rapid SMR and somatic synthesis requirements.

Emergent somatic growth trajectories (Fig 6D) were similar to the age-structured growth patterns calculated in the federal stock assessment for Gulf of Mexico King Mackerel [37]. The logarithmic fit to the simulated length-at-age scatter tracked the back-calculated von Bertalanffy curve (blue line) with high fidelity: simulated agents reached an average fork length of 850mm by age 5 and asymptotically approached their maximum size parameter by age 15–18. This growth curve emerged naturally from the energetic balance remaining after metabolic and reproductive costs, showing that the model's bioenergetic allocation rules scale up to produce realistic demographic structures. Although the fitted logarithmic curve from the model rests below the assessment growth rate, the model clearly demonstrates variability among the agent growth that encompasses this benchmark. Further, the population was exposed to high variance in mortality (Fig 6E) with the largest increases in mortality occurring for starvation mortality during peak spawning seasons, a result of large amounts of larvae being removed from the simulation because of density-dependent factors. The bioenergetics functionality additionally creates Beverton-Holt fits to estimate a steepness of 0.45 based on the model configuration and egg production (Fig A in S1 Appendix).

### Population dynamics

The King Mackerel simulation was long-term (60 years) and contained multiple species, allowing for an examination of the species’ population dynamics. At the population level, the cumulative behavior and physiology of individual agents produced stable, multi-decadal demographic trends (Fig 7). Total abundance and biomass exhibited a partially decoupled relationship, where biomass lagged abundance fluctuations by approximately 3 years. Seasonal spawning events triggered rapid, high-amplitude spikes in total abundance (up to +45%), which then decayed over the year as high larval and juvenile mortality took effect. In contrast, total population biomass remained highly buffered, fluctuating within a much narrower (~20%) band, as the cohort-level weight gain of surviving individuals offset the rapid loss of juvenile numbers. This buffering demonstrates that the model correctly reproduces the biomass storage capacity of long-lived, age-structured populations. Size-frequency distributions sampled across the simulation illustrated the progression of cohort-level demographic transitions (Fig 7B). In the early simulation period (Winter 1986), the population was dominated by large, unharvested adult cohorts centered near 1000mm. As the simulation progressed, the overall pattern (large number of larvae and young adults) remained the same. This pattern is indicative of a population that has high larval and early juvenile mortality (via predation and starvation) and fishing pressure that targets large adults. There was a seasonal difference in the proportion of the population that was larvae, which corresponded to the peak spawning seasons (late summer). Mean individual length (Fig 7C) fluctuated inversely with these recruitment pulses, dropping sharply from 450mm to 200mm during high-recruitment years, and recovering as cohorts aged, demonstrating a highly dynamic demographic age structure. In terms of performance, this 60-year King Mackerel run (plus a 25-year spin-up; 4,420 one-week timesteps) completed in approximately 4 hours on a standard consumer laptop (16 CPUs, 2 GPUs, 32 GB RAM), and would scale further on cloud or HPC resources.

**Fig 7:**
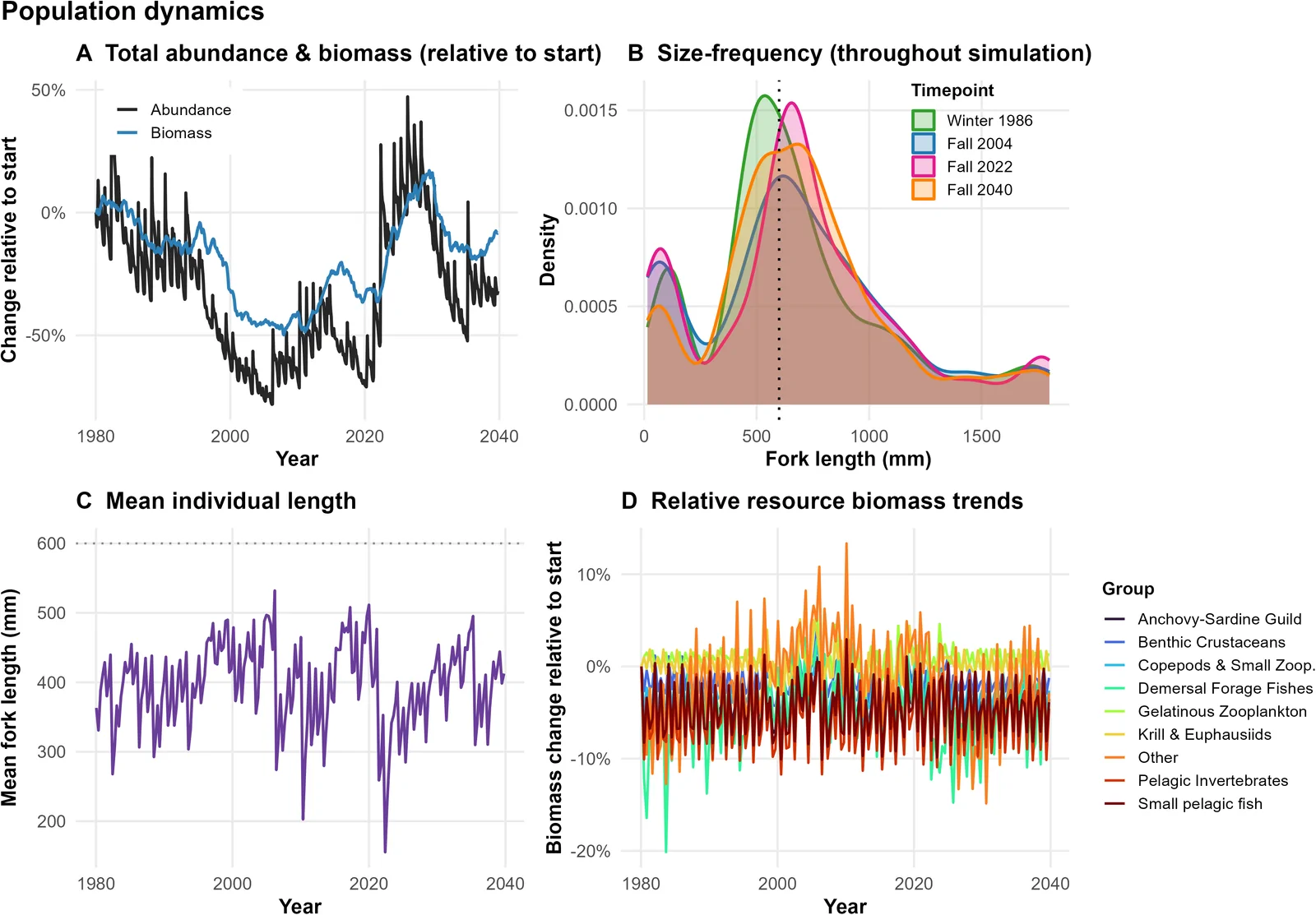
Population-level demographic and multi-trophic dynamics over the simulation. **A)** Relative change in total abundance (black line) and total biomass (blue line) over time, scaled on a single percentage axis relative to the initial state. **B)** Size-frequency density plots of the King Mackerel population sampled at four distinct multi-decadal time slices, showing the evolution of age structure. **C)** Spatially integrated mean individual fork length (mm) over time, showing seasonal fluctuations linked to spawning and recruitment. **D)** Relative change in biomass over time for nine major mid-trophic resource groups, illustrating long-term trophic stability.

Mid-trophic resource group biomasses also exhibited stable, non-collapsing oscillations within a ~ 15% bound (Fig 7D). This long-term stability indicates that the bottom-up logistic growth of the resources and the top-down grazing pressure from the mackerel agents achieved a stable multi-trophic equilibrium. Seasonal primary production (NPP) forcing drove resource biomass peaks during spring and summer, which were then grazed down in the autumn, demonstrating that *SwimmingIndividuals* can sustain complex, multi-trophic feedback loops over multi-decadal timelines without structural collapse.

### Fisheries impacts

The implementation of five distinct fishing fleets generated space- and size-structured catch and mortality profiles (Fig 8). Total fishing mortality (F, Fig 8A) on the adult mackerel population rose by approximately 60% during the middle decade of the simulation, corresponding to high stock biomass and high quota allocations, before stabilizing near the baseline toward the end of the run. This long-term trend was overlaid by high-amplitude seasonal oscillations driven by regulatory fleet season openings and winter migration-induced changes in fleet catchability. The mean catch length of a fleet demonstrated sharp size-selectivity separation across the five fleets, arising directly from their parameterized gear selectivities (Fig 8B). The commercial gillnet fleet caught small individuals tightly centered near 750mm due to its narrow, dome-shaped selectivity curve, whereas commercial hook-and-line and recreational fleets harvested larger adult size classes exceeding 1000mm due to their logistic and knife-edge selectivities. Annual relative landings showed distinct, fleet-specific responses to regulatory quota constraints (Fig 8C). Recreational catches exhibited high interannual variability (~40%) linked to seasonal fish availability, while commercial fleets reached their annual quotas with high consistency, demonstrating the model's capacity to represent complex regulatory and spatial fleet dynamics.

**Fig 8:**
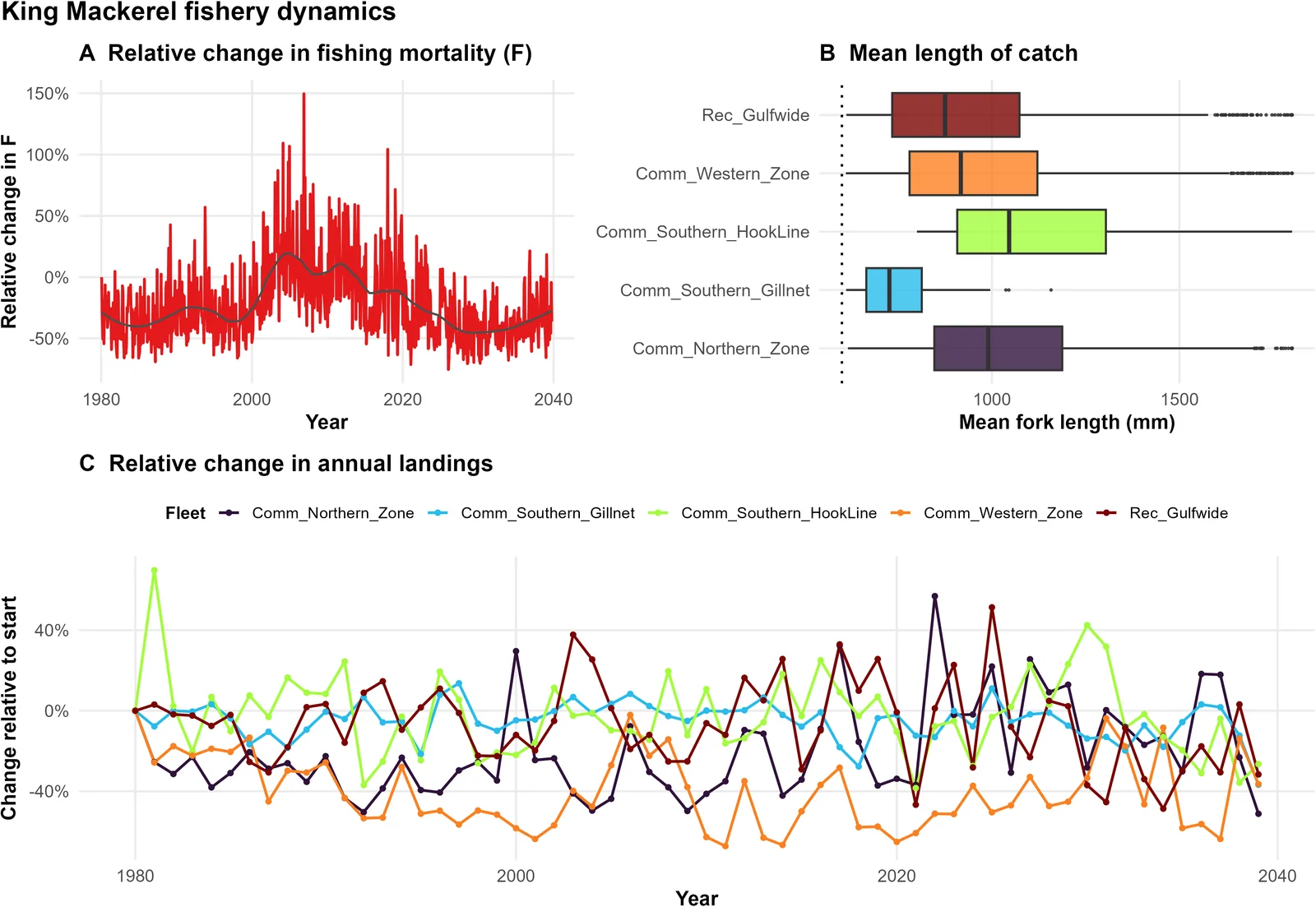
Multi-fleet fishery and harvest dynamics. **A)** Relative change in total fishing mortality (F) on adult King Mackerel over the simulation (red line), with a smoothed loess trendline (grey line). **B)** Boxplots of the fork length (mm) of harvested mackerel across the five active fishing fleets, illustrating gear-specific size selectivity (dotted vertical line indicates the length at maturity [600m]). **C)** Relative change in annual landings (metric tons) over time for each fleet, scaled on a single percentage axis relative to their initial catches.

## Discussion

### Ecological and management implications

The capacity of *SwimmingIndividuals* to resolve fine-scale, multi-taxon physiological states across broad three-dimensional domains offers unique advantages for ecosystem-based fisheries management (EBFM) and operational Management Strategy Evaluation (MSE). Traditional bulk-biomass modeling tools often aggregate species into unstructured biomass pools, which can mask the crucial role of individual-level size structures and energetic buffers in dictating cohort survival under high harvest levels [3,5,31]. By tracking discrete, age-structured superindividuals that allocate net surplus energy dynamically among physiological maintenance, somatic growth, and reproduction, *SwimmingIndividuals* preserves the underlying phenotypic and spatial heterogeneity necessary to evaluate cumulative ecosystem risks [36,45,46]. Under this framework, macro-ecological patterns (such as density-dependent compensation, recruitment patterns, and fleet-specific size selectivities) are not hardcoded but emerge from localized energetic optimization [3,39].

By linking thermodynamic maintenance and hunting behaviors to spatial-temporal oceanographic fields, the model's spatial predation and natural mortality maps become highly sensitive to environmental stressors. Rather than utilizing static, geographically uniform natural mortality coefficients (*M*) or pre-allocated dietary matrices, biomass transfer in our framework is regulated by dynamic physical-biological overlaps [47]. Shifts in physical fields (e.g., ocean warming, salinity anomalies, changes in regional primary production) immediately modify both the piece-wise trapezoidal habitat capacity arrays and the first-principles visual detection envelopes of simulated predators [2,33]. This physical-biological coupling makes the framework exceptionally well-suited for simulating the spatial and structural impacts of climate change on trophic networks, and for projecting the efficacy of spatial conservation measures, such as Marine Protected Areas (MPAs) or dynamic seasonal closures, under non-analog environmental baselines [5,6].

### Performance and scalability

The computational bottlenecks that have historically restricted agent-based frameworks to highly simplified behavioral rules or localized grids are aided here through a hybrid CPU/GPU architecture. High-performance computing (HPC) constraints in ecology frequently force models to choose between detailed, multi-species bioenergetic logic and large spatial scales, which often results in the oversimplification of horizontal movement or visual searching patterns [3,7]. By implementing parallelized GPU kernels for memory-intensive operations (such as spatial neighborhood lookups, visual range evaluations, and fleet quota removals) while retaining threaded CPU execution for sequential, branch-heavy physiological and pathfinding calculations, these biological processes can be simulated at large spatial scales.

This parallel division of labor is further optimized by our thread-safe path memorization engine. Rather than executing redundant, computationally demanding A* pathfinding sweeps for every overlapping agent at each timestep, paths along the local capacity gradient are memorized and shared among identical cohorts within the same cell [12]. By bounding the maximum horizontal displacement of agents to a daily swimming cap, the model preserves cohesive, seasonal migration corridors along coastal shelf breaks and topographic boundaries, enabling the model to capture the fine-scale spatial overlaps that dictate both predator-prey encounter rates and commercial fleet catchability.

## Availability and future directions

*SwimmingIndividuals* v1.0 is available in a public Github repository (https://github.com/mattwoodstock/SwimmingIndividuals). The current formulation of behavioral vertical movements in *SwimmingIndividuals* v1.0 represents a target for future development. At present, diel vertical migration (DVM) and cetacean diving archetypes operate as stochastically drawn, archetype-specific depth overrides that operate independently of local predator and prey fields [27,28]. Integrating a fully active, state-dependent decision engine, where simulated agents choose their depth coordinate dynamically to maximize a multi-objective fitness function that balances light-dependent predation risk against local prey encounter rates, would allow complex, adaptive vertical profiles to emerge [8,26,48]. Under such a game-theoretic vertical model, migrations would adapt dynamically to changing ocean clarity, predator densities, and lunar cycles, providing a more robust framework for evaluating the ecological impacts of mesopelagic harvesting and deep-sea mineral extraction [29,48]. Relatedly, replacing the current spatially uniform diel light curve with a seasonally and spatially varying light–dark cycle (e.g., a latitude- and date-dependent photoperiod modulated by local turbidity) is a possible extension that would sharpen the light-driven components of both visual predation and vertical migration.

Similarly, the recruitment submodel can be expanded by integrating biophysical coupling with hydrodynamic transport fields. While our current reproductive module models density-dependent egg-to-recruit survival on a static spatial grid, it currently has two limitations: 1) it does not include alternative stock-recruitment relationships (e.g., Ricker model [49]), and 2) it does not explicitly track the passive transport of early life stages by marine currents [40]. Linking the spawning output of mature agents to a Lagrangian particle tracking or Eulerian hydrodynamic transport model (e.g., ROMS; *Oceananigans.jl* [50]) represents a critical next step. This biophysical integration would allow researchers to model how larval drift, regional temperature anomalies, and current shearing affect population connectivity, recruitment success, and the establishment of local spawning-stock-biomass thresholds under shifting oceanographic currents [37,40,51].

Finally, expanding the fishing fleet module from static, geographically bounded harvest grids to adaptive, agent-based vessel objects would greatly enhance the model's socio-ecological utility. Standard fisheries modules evaluate removals probabilistically based on uniform regional quotas, but do not capture how fishers adapt their spatial behavior to changing fuel costs, market prices, and weather [29,52]. Constructing a fully integrated bio-economic framework, where virtual vessels forage for fish schools using their own search heuristics, travel budgets, and regulatory constraints, would provide a more realistic platform for Management Strategy Evaluation (MSE) [29]. Linking these human behavioral dynamics with emerging deep learning techniques for ecological parameterization and scenario planning would enhance *SwimmingIndividuals* ability to be an open-source tool to create “digital twins” for predictive marine science and resilient ecological management [53].

## Supporting information

S1 Appendix Supplementary Figures and Tables. (DOCX)
