# Supplementary material for: SwimmingIndividuals: A high-performance agent-based model for marine ecosystems
Source: PLoS Comput Biol. 2026 Nov 6;22(11):e1013610. doi: 10.1371/journal.pcbi.1013610 (PMC13766604; doi:10.1371/journal.pcbi.1013610)
Supplement: Supplementary file 1 [file pcbi.1013610.s001.docx]

**Supplemental Material: *SwimmingIndividuals* model input parameters**

*King Mackerel (Scomberomorus cavalla), Gulf of America case study*

This supplement documents every user-supplied input parameter required to run the *SwimmingIndividuals* agent-based model, including units and definitions. Values shown are those used in the King Mackerel (*Scomberomorus cavalla*) case study presented in the main text. Equation numbers cross-reference the model description in the Methods.

Inputs are supplied as plain-text CSV files plus gridded environmental drivers, resolved through a master manifest (*files.csv*; S1 Table). S2 and S3 Tables give the simulation control and spatial domain settings. S4-S9 Tables describe the biological entities: focal species traits, mid-trophic resource groups, environmental preferences and vertical distributions. S10 and S11 Tables define the fishing fleets, and S12-S14 Tables give the seasonal and recruitment forcing.

The simulation used a 10,080-minute (weekly) timestep for 4,420 timesteps, of which the first 1,300 (24.9 yr) were discarded as spin-up, giving the 59.8-year analysis period reported in the main text. Outputs were written every 43,200 minutes (30 d). The other scenarios had similar input requirements, but are not displayed here, as they simply had different inputs representative of analog species and archetypes.

**Table A. Input file manifest.**

*Source file: files.csv*

*SwimmingIndividuals* resolves every input through a master manifest. Each key below points to a file or directory within the scenario folder.

| **Key** | **Destination** | **Contents** |
| --- | --- | --- |
| scen_dir | examples/Mackerel | Root directory of the scenario; all relative paths below resolve against it. |
| res_dir | results | Directory into which model outputs are written. |
| focal_trait | traits.csv | Focal species biological and behavioural traits (S4 Table). |
| resource_trait | resource_traits.csv | Resource group traits (S5, S6Tables). |
| focal_z_dist_night | z_distributions_night.csv | Night-time depth distribution of focal species (S9 Table). |
| focal_z_dist_day | z_distributions_day.csv | Daytime depth distribution of focal species (S9 Table). |
| grid | grid.csv | Spatial domain definition (S3 Table). |
| params | params.csv | Simulation control parameters (S2 Table). |
| resource_z_dist_night | z_pool_distributions_night.csv | Night-time depth distributions of resource groups (S9 Table). |
| resource_z_dist_day | z_pool_distributions_day.csv | Daytime depth distributions of resource groups (S9 Table). |
| fisheries | fishery.csv | Fishing fleet definitions and regulations (S10, S11 Tables). |
| envi_pref | envi_pref.csv | Environmental preference and tolerance bounds per species and variable (S7, S8 Tables). |
| reproduction | reproduction.csv | Monthly spawning seasonality multipliers (S12 Table). |
| growth_seasonality | growth_seasonality.csv | Monthly resource growth multipliers (S13 Table). |
| env_xml | environment.xml | XML manifest describing the file paths to gridded environmental driver layers. |
| env_dir | EnvData | Directory holding the environmental raster driver files. The directory should have subdirectories with the names of each driver. |
| recruitment_env | recruitment_env.csv | Environmental and density-dependent recruitment parameters (S14 Table). |

**Table B. Simulation control parameters.**

*Source file: params.csv*

Global settings controlling run length, temporal resolution, output frequency and hardware target. The values in this table align with the King Mackerel simulation.

| **Parameter** | **Value** | **Units** | **Description** |
| --- | --- | --- | --- |
| numspec | 1 | species | Number of focal species. |
| numresource | 17 | groups | Number of resource groups. |
| nts | 4420 | timesteps | Number of Time Steps model and spinup. Total timesteps including spin-up; 4,420 × 10,080 min = 84.8 yr. |
| spinup | 5,241,600 | min | Number of minutes of the spinup. 5,2,41,600 min = 10 yr; results are not saved. |
| model_dt | 10,080 | min | Number of minutes between time steps. 10,080 min = 7 d (weekly timestep). This is dt in Eqs 3, 5, 7, 8, 24, 26 and 27. |
| n_iter | 1 | iterations | Number of model iterations to run. |
| output_size_bins | 10 | bins | Number of size bins for species' outputs (only applies to arrays). |
| output_dt | 43,200 | min | Interval between Timestep Outputs. 43,200 min = 30 d. |
| num_foraging_attempts | 100 | attempts | Maximum number of foraging attempts to cycle through. Includes tradeoff between realism and performance, but a high number will almost never be reached because of time and stomach fullness constraints. |
| plt_diags | 1 | flag (0/1) | 0 = Do not plot diagnostics : 1 = plot diagnostics. |
| start_year | 1980 | calendar year | Post-spinup calendar year the simulation begins (drives dates + environmental forcing). |
| start_month | 1 | month (1–12) | Optional start month (default 1 if omitted). |
| start_day | 1 | day of month | Optional start day (default 1 if omitted). |

**Table C. Spatial domain definition.**

*Source file: grid.csv*

The domain is defined both in projected metres (extent) and geographic degrees (corner coordinates). The grid in the current model spans 131 × 53 cells of 0.1336°, i.e. approximately 1,600 km × 783 km covering the Gulf of America. The grid must match the resolution of the environmental driver rasters.

| **Parameter** | **Value** | **Units** | **Description** |
| --- | --- | --- | --- |
| depthmax | 1000 | m | Maximum Depth (insert positive value) |
| latres | 53 | cells | Number of latitude grid cells |
| lonres | 131 | cells | Number of Longitude Grid Cells |
| depthres | 1 | cells | Number of depth Grid Cells |
| cellsize | 0.13 | decimal degrees | Size of the cell degrees |
| xurcorner | -80 | decimal degrees | Upper left longitude |
| xllcorner | -98 | decimal degrees | Lower left longitude |
| yulcorner | 31 | decimal degrees | Upper left latitude |
| yllcorner | 23.93 | decimal degrees | Lower left latitude |

**Table D. Focal species traits.**

*Source file: traits.csv*

One row per focal species. Values shown are those used in the King Mackerel case study.

| **Parameter** | **Value** | **Units** | **Description** | **Source** |
| --- | --- | --- | --- | --- |
| SpeciesLong | King Mackerel | — | Full species name; used to match this species across all other input files. | — |
| SpeciesShort | King | — | Short label used in output file names. | — |
| Type | non_mig | — | Behavioural archetype governing vertical movement: non_mig (habitat-seeking only), dvm_strong, dvm_weak, or pelagic_diver. | This study (model configuration) |
| Taxa | 1 | integer code | Taxonomic group identifier used to group species in summary outputs. 1 = fish, 2 = crustacean, 3 = cephalopod. Functionally only used in the deep-sea metabolic rate equation | — |
| MR_type | 1 | integer code | Metabolic archetype; selects which respiration formulation is applied (Eqs 5–9). 1 = default ectotherm, 2 = cetacean, 3 = deep-sea | This study (model configuration) |
| Biomass | 0.2 | mt km^-2^ | Target biomass density used to seed the initial agent population across the domain. | SEDAR (2014) |
| Min_Size | 15 | mm | Minimum body length in the initial size distribution. | This study (initialization) |
| Max_Size | 1,800 | mm | Maximum (asymptotic) body length; caps somatic growth and sets the reproductive allocation scale *L_max_* (Eqs 13, 16). | SEDAR (2014); FishBase |
| Max_age | 25 | yr | Maximum age *A_max_*; agents are removed once age reaches this value (senescence, Eq 17). | SEDAR (2014); FishBase |
| LWR_a | 0.00376 | g cm^-1^ | Coefficient of the length–weight relationship *W = a(L/10)^b^*, with *L* in mm (Eq 16). | SEDAR (2014); FishBase |
| LWR_b | 3.07 | dimensionless | Exponent of the length–weight relationship. | SEDAR (2014); FishBase |
| Swim_velo | 1.5 | body lengths s^-1^ | Sustained swimming speed used to convert available time into distance moved during habitat-seeking. | This study |
| Surface_Interval | 0 | min | Time spent at the surface between dives (pelagic_diver archetype only; 0 if unused). | Not applicable (non-diving archetype) |
| Dive_Interval | 0 | min | Time spent at depth per dive (pelagic_diver archetype only; 0 if unused). | Not applicable (non-diving archetype) |
| Dive_Min_Day | 0 | m | Shallowest daytime dive depth (pelagic_diver only). | Not applicable (non-diving archetype) |
| Dive_Max_Day | 0 | m | Deepest daytime dive depth (pelagic_diver only). | Not applicable (non-diving archetype) |
| Dive_Min_Night | 0 | m | Shallowest night-time dive depth (pelagic_diver only). | Not applicable (non-diving archetype) |
| Dive_Max_Night | 0 | m | Deepest night-time dive depth (pelagic_diver only). | Not applicable (non-diving archetype) |
| Min_Prey | 0.01 | dimensionless | Lower bound of the prey-to-predator body-length ratio defining the ingestible prey window. | This study (predator–prey size window) |
| Max_Prey | 0.5 | dimensionless | Upper bound of the prey-to-predator body-length ratio. | This study (predator–prey size window) |
| Energy_density | 7,000 | J g^-1^ | Energy density of body tissue; converts between biomass and energy throughout the bioenergetics (Eqs 11, 16). | Hanson et al. (1997); published fish energy-density values |
| Handling_Time | 0.1 | min | Prey handling time *t_h_* in the Holling Type II functional response (Eq 26). | This study (functional-response parameter) |
| Max_Stomach_a | 0.25 | dimensionless | Coefficient of maximum stomach capacity, expressed as a proportion of body mass: *capacity = a·W^b^*. | This study (model assumption) |
| Max_Stomach_b | 0.67 | dimensionless | Mass exponent of maximum stomach capacity. | This study (model assumption) |
| Sex_Ratio | 0.5 | proportion | Fraction of spawning individuals that are female (Eq 28). | Assumed 1:1 (this study) |
| Larval_Size | 15 | mm | Body length assigned to newly recruited agents. | This study (initialization) |
| Larval_Duration | 14 | d | Larval period before recruits enter the modelled population. | This study |
| Hatch_Survival | 0.01 | proportion | Fraction of eggs surviving to recruitment (Eq 28). | This study (calibrated) |
| School_Size | 100,000 | individuals | Maximum number of individuals represented by a single superindividual agent; caps school consolidation. | This study (superindividual cap) |
| L_mat | 600 | mm | Length at maturity *L_mat_*; agents mature once body length reaches this value. | SEDAR (2014); FishBase |
| Resp_a | 0.6 | dimensionless | Coefficient of the standard metabolic rate relationship (Eq 5); SMR is returned in mg O_2_ g^-1^ d^-1^ before conversion to joules. | Hanson et al. (1997); Hewett & Johnson (1987) |
| Resp_b | -0.25 | dimensionless | Body-mass exponent of standard metabolic rate (Eq 5). | Hanson et al. (1997); Hewett & Johnson (1987) |
| Resp_q | 0.069 | °C^-1^ | Temperature coefficient of standard metabolic rate (Eq 5). | Hanson et al. (1997); Hewett & Johnson (1987) |
| Activity_Mult | 2.5 | dimensionless | Multiplier applied to standard metabolic rate during active periods to give active metabolic rate (Eqs 6, 9). | Hanson et al. (1997) |
| M | 0.05 | yr^-1^ | Annual instantaneous natural mortality rate applied per agent per timestep that is not already included in the model (Eq 24). | This study (residual; cf. SEDAR (2014), M ≈ 0.17 yr⁻¹) |

**Sources for S4 Table.** Life-history values follow the Gulf of Mexico King Mackerel benchmark assessment and standard species compilations; metabolic parameters follow the Wisconsin bioenergetics framework. Full citations: *SEDAR (2014)* = SEDAR 38 Stock Assessment Report: Gulf of Mexico King Mackerel. Southeast Data, Assessment, and Review, North Charleston, SC. *FishBase* = Froese, R. and D. Pauly, editors. FishBase (Scomberomorus cavalla). www.fishbase.org. *Hanson et al. (1997)* = Hanson, P.C., T.B. Johnson, D.E. Schindler, and J.F. Kitchell. Fish Bioenergetics 3.0. University of Wisconsin Sea Grant Institute. *Hewett & Johnson (1987)* = Hewett, S.W. and B.L. Johnson. A Generalized Bioenergetics Model of Fish Growth for Microcomputers. University of Wisconsin Sea Grant Technical Report WIS-SG-87-245. “This study” denotes values set as model configuration, initialization, or calibration choices rather than taken from a published source; these should be confirmed or replaced with the primary source used where one exists.

**Table E. Mid-trophic resource group parameters.**

*Source file: resource_traits.csv*

Column definitions for the resource groups in a *SwimmingIndividuals* model. Group-specific values are given in S6 Table.

| **Parameter** | **Units** | **Description** |
| --- | --- | --- |
| SpeciesLong | — | Name of the mid-trophic resource group. |
| Min_Size | mm | Minimum body length of individuals in the group. |
| Max_Size | mm | Maximum body length of individuals in the group. |
| Biomass | mt km^-2^ | Initial biomass density seeded to each grid cell, scaled by the habitat capacity map. |
| LWR_a | g cm^-2^ | Coefficient of the length–weight relationship for the group. |
| LWR_b | dimensionless | Exponent of the length–weight relationship. |
| Growth | yr^-1^ | Intrinsic rate of increase used in the logistic growth solution (Eq 25), before monthly seasonal multipliers. |
| Z | yr^-1^ | Annual instantaneous background mortality rate not otherwise represented in the model (M_R_ in Eq 27). |
| Energy_density | J g^-1^ | Energy density of group tissue; sets the energy delivered per gram consumed. |
| Min_Prey | dimensionless | Lower bound of the prey-to-predator length ratio for this group when it forages; 0 for non-predatory groups. |
| Max_Prey | dimensionless | Upper bound of the prey-to-predator length ratio; 0 for non-predatory groups. |
| Daily_Ration | proportion of body mass d^-1^ | Maximum daily ration; caps realised consumption in the functional response (Eq 26). |
| Handling_Time | min | Prey handling time *t_h_* for this group (Eq 26). |

**Table F. Mid-trophic resource group parameter values.**

*Source file: resource_traits.csv*

Parameter values for all 17 resource groups. See S5 Table for units and definitions.

| **SpeciesLong** | **Min**  **Size** | **Max**  **Size** | **Biomass** | **LWR_a** | **LWR_b** | **Growth** | **Z** | **Energy**  **Density** | **Min**  **Prey** | **Max Prey** | **Daily**  **Ration** | **Handling Time** |
| --- | --- | --- | --- | --- | --- | --- | --- | --- | --- | --- | --- | --- |
| Copepods & Small Zoop. | 0.1 | 2 | 4000 | 5.00E-03 | 3 | 45.0 | 1.5 | 4200 | 0 | 0 | 0.1 | 1 |
| Krill & Euphausiids | 2 | 10 | 4500 | 5.00E-03 | 3 | 15.0 | 1.2 | 4500 | 0 | 0 | 0.1 | 1 |
| Gelatinous Zooplankton | 1 | 20 | 120 | 2.00E-03 | 2.8 | 15.0 | 0.8 | 600 | 0 | 0 | 0.1 | 1 |
| Anchovy-Sardine Guild | 3 | 200 | 15 | 5.00E-03 | 3.1 | 30.0 | 0.4 | 6500 | 0.001 | 0.07 | 0.06 | 1 |
| Small pelagic fish | 10 | 300 | 10 | 6.00E-03 | 3 | 30.0 | 0.4 | 6800 | 0.001 | 0.05 | 0.06 | 1 |
| Pelagic Invertebrates | 1 | 100 | 20 | 8.00E-03 | 2.9 | 20.0 | 0.5 | 4100 | 0.05 | 0.14 | 0.05 | 1 |
| Benthic Crustaceans | 10 | 150 | 8 | 9.00E-03 | 2.9 | 25.0 | 0.8 | 4200 | 0 | 0 | 0.08 | 1 |
| Demersal Forage Fishes | 30 | 350 | 6 | 1.10E-02 | 3 | 20.0 | 0.5 | 5500 | 0.02 | 0.15 | 0.05 | 1 |
| Spanish Mackerel | 100 | 800 | 0.15 | 7.00E-03 | 3 | 20.5 | 0.1 | 6200 | 0.08 | 0.2 | 0.06 | 1 |
| Other Mackerel | 80 | 600 | 0.15 | 6.50E-03 | 3 | 20.5 | 0.1 | 6100 | 0.08 | 0.2 | 0.06 | 1 |
| Cephalopods | 10 | 500 | 2.5 | 1.20E-02 | 2.8 | 20.5 | 0.15 | 4300 | 0.05 | 0.15 | 0.05 | 1 |
| Small tunas | 150 | 1000 | 0.1 | 9.00E-03 | 3 | 20.2 | 0.08 | 6900 | 0.1 | 0.25 | 0.05 | 1 |
| Pelagic fishes | 200 | 1500 | 0.5 | 1.00E-02 | 3 | 20.0 | 0.05 | 7200 | 0.12 | 0.3 | 0.04 | 1 |
| Medium-sized fishes | 50 | 500 | 3.0 | 8.50E-03 | 3.1 | 20.5 | 0.1 | 5800 | 0.03 | 0.18 | 0.05 | 1 |
| Other Large Sharks | 1500 | 5500 | 0.002 | 8.00E-03 | 3.0 | 2.5 | 0.1 | 5500 | 0.15 | 0.6 | 0.01 | 10 |
| Large coastal pelagics | 500 | 2000 | 0.005 | 9.00E-03 | 3.0 | 5.0 | 0.1 | 6500 | 0.45 | 0.8 | 0.01 | 10 |
| Cetaceans | 2000 | 9000 | 0.002 | 1.50E-02 | 3.0 | 2.5 | 0.05 | 8000 | 0.10 | 0.4 | 0.01 | 10 |

**Table G. Environmental preference parameters.**

*Source file: envi_pref.csv*

Four bounds per species per environmental variable define the piecewise-linear trapezoidal habitat suitability function *H(V)* (Eqs 18a–d).

| **Parameter** | **Units** | **Description** |
| --- | --- | --- |
| species | — | Species or resource group to which the preference applies. |
| variable | — | Environmental variable scored (e.g., temp-surf (°C), salinity (PSU), or bathymetry (m)). |
| pref_min | variable units | Lower bound of tolerated habitat; suitability *H(V)* = 0 at or below this value (Eq 18a). |
| opt_min | variable units | Lower bound of optimal habitat; suitability rises linearly from pref_min to opt_min (Eq 18b). |
| opt_max | variable units | Upper bound of optimal habitat; suitability = 1 between opt_min and opt_max (Eq 18c). |
| pref_max | variable units | Upper bound of tolerated habitat; suitability declines linearly from opt_max to pref_max and is 0 beyond (Eqs 18a, 18d). |

**Table H. Environmental preference values.**

*Source file: envi_pref.csv*

Preference bounds for every species and resource group. Units follow the variable: temp-surf in °C, salinity in PSU, bathymetry in m.

| **species** | **variable** | **pref_min** | **opt_min** | **opt_max** | **pref_max** |
| --- | --- | --- | --- | --- | --- |
| King Mackerel | temp-surf | 22 | 25 | 29 | 31 |
|  | bathymetry | 0 | 0 | 150 | 250 |
|  | salinity | 28 | 32 | 36 | 38 |
| Copepods & Small Zoop. | temp-surf | 12 | 18 | 28 | 34 |
|  | bathymetry | 0 | 1 | 400 | 500 |
|  | salinity | 24 | 30 | 36 | 38 |
| Krill & Euphausiids | temp-surf | 10 | 16 | 26 | 32 |
|  | bathymetry | 0 | 1 | 400 | 800 |
|  | salinity | 26 | 32 | 36 | 38 |
| Gelatinous Zooplankton | temp-surf | 14 | 20 | 30 | 35 |
|  | bathymetry | 0 | 1 | 400 | 600 |
|  | salinity | 25 | 30 | 36 | 38 |
| Anchovy-Sardine Guild | temp-surf | 16 | 21 | 29 | 33 |
|  | bathymetry | 0 | 1 | 400 | 600 |
|  | salinity | 27 | 32 | 36 | 38 |
| Small pelagic fish | temp-surf | 18 | 22 | 30 | 34 |
|  | bathymetry | 0 | 1 | 400 | 600 |
|  | salinity | 28 | 33 | 36 | 38 |
| Pelagic Invertebrates | temp-surf | 12 | 17 | 28 | 33 |
|  | bathymetry | 0 | 1 | 400 | 1000 |
|  | salinity | 30 | 34 | 37 | 38 |
| Benthic Crustaceans | temp-surf | 15 | 20 | 28 | 32 |
|  | bathymetry | 0 | 2 | 100 | 200 |
|  | salinity | 15 | 25 | 36 | 38 |
| Demersal Forage Fishes | temp-surf | 16 | 21 | 28 | 32 |
|  | bathymetry | 0 | 5 | 150 | 250 |
|  | salinity | 20 | 28 | 36 | 38 |
| Other Mackerel | temp-surf | 20 | 24 | 31 | 34 |
|  | bathymetry | 0 | 25 | 200 | 400 |
|  | salinity | 30 | 34 | 37 | 38 |
| Cephalopods | temp-surf | 15 | 18 | 28 | 32 |
|  | bathymetry | 20 | 50 | 300 | 500 |
|  | salinity | 28 | 33 | 36 | 38 |
| Spanish Mackerel | temp-surf | 19 | 23 | 31 | 35 |
|  | bathymetry | 0 | 10 | 100 | 200 |
|  | salinity | 26 | 30 | 36 | 38 |
| Small tunas | temp-surf | 20 | 24 | 30 | 33 |
|  | bathymetry | 50 | 200 | 1000 | 4000 |
|  | salinity | 30 | 33 | 36 | 38 |
| Pelagic fishes | temp-surf | 21 | 25 | 31 | 34 |
|  | bathymetry | 0 | 200 | 1000 | 4000 |
|  | salinity | 28 | 32 | 36 | 38 |
| Medium-sized fishes | temp-surf | 15 | 20 | 28 | 32 |
|  | bathymetry | 0 | 10 | 120 | 200 |
|  | salinity | 22 | 28 | 36 | 38 |
| Oceanic pelagics | temp-surf | 21 | 24 | 29 | 32 |
|  | bathymetry | 50 | 200 | 1000 | 4000 |
|  | salinity | 30 | 34 | 37 | 38 |
| Billfishes | temp-surf | 22 | 25 | 29 | 31 |
|  | bathymetry | 50 | 200 | 1000 | 4000 |
|  | salinity | 31 | 34 | 37 | 38 |
| Tunas | temp-surf | 20 | 23 | 29 | 32 |
|  | bathymetry | 50 | 200 | 1000 | 4000 |
|  | salinity | 31 | 34 | 37 | 38 |
| Pelagic sharks | temp-surf | 19 | 23 | 30 | 34 |
|  | bathymetry | 10 | 50 | 500 | 1200 |
|  | salinity | 28 | 32 | 36 | 38 |
| Other Large Sharks | temp-surf | 18 | 22 | 32 | 35 |
|  | bathymetry | 0 | 2 | 50 | 150 |
|  | salinity | 5 | 15 | 35 | 38 |
| Large coastal pelagics | temp-surf | 22 | 25 | 31 | 34 |
|  | bathymetry | 0 | 5 | 80 | 150 |
|  | salinity | 28 | 32 | 36 | 38 |
| Cetaceans | temp-surf | 15 | 20 | 32 | 36 |
|  | bathymetry | 0 | 5 | 100 | 400 |
|  | salinity | 20 | 28 | 36 | 38 |

**Table I. Vertical (depth) distribution parameters.**

*Source file: z_distributions_day.csv, z_distributions_night.csv, z_pool_distributions_day.csv, z_pool_distributions_night.csv*

Four files supply the tri-modal Gaussian mixture from which target depths are drawn at each diel transition (Eq 19): separate day and night files for focal species and for resource groups.

| **Parameter** | **Units** | **Description** |
| --- | --- | --- |
| SpeciesLong | — | Species or resource group the distribution applies to. |
| SpeciesShort | — | Short label for the group. |
| Type | — | Record type; "Distribution" marks a depth-distribution row. |
| mu1 | m | Mean depth of the first Gaussian component (Eq 19). |
| mu2 | m | Mean depth of the second Gaussian component. |
| mu3 | m | Mean depth of the third Gaussian component. |
| sigma1 | m | Standard deviation of the first component. |
| sigma2 | m | Standard deviation of the second component. |
| sigma3 | m | Standard deviation of the third component. |
| lambda1 | proportion | Mixing weight of the first component; weights sum to 1 (Eq 19). |
| lambda2 | proportion | Mixing weight of the second component. |
| lambda3 | proportion | Mixing weight of the third component. |

**Table J. Fishery parameters.**

*Source file: fishery.csv*

One row per fleet. Fleet-specific values are given in S11 Table.

| **Parameter** | **Units** | **Description** |
| --- | --- | --- |
| FisheryName | — | Unique fleet identifier used in catch and effort outputs. |
| Species | — | Species affected by this fleet. |
| Role | — | Whether the species is the fleet target or bycatch; only target catch counts against the quota. |
| SelectivityType | — | Gear selectivity form: logistic (Eq 20), knife_edge (Eq 21), or dome_shaped (Eq 22). |
| L50 | mm | Length at 50% selectivity (ascending limb for dome-shaped gear). |
| Slope | mm^-1^ | Steepness of the logistic selectivity curve (ascending limb). |
| L50_2 | mm | Length at 50% selectivity on the descending limb (dome-shaped gear only; 0 if unused). |
| Slope_2 | mm^-1^ | Steepness of the descending limb (dome-shaped gear only; 0 if unused). |
| Quota | mt yr^-1^ | Annual catch quota; resets on 1 January of each simulated year. |
| StartDay | day of year | First day of the open season. |
| EndDay | day of year | Last day of the open season. |
| XMin | decimal degrees | Western longitude bound of the fishing area. |
| XMax | decimal degrees | Eastern longitude bound of the fishing area. |
| YMin | decimal degrees | Southern latitude bound of the fishing area. |
| YMax | decimal degrees | Northern latitude bound of the fishing area. |
| ZMin | m | Shallowest depth fished. |
| ZMax | m | Deepest depth fished. |
| SlotMin | mm | Minimum retainable length; agents below this are not removed. |
| SlotMax | mm | Maximum retainable length. |
| BagLimit | fish vessel^-1^ d^-1^ | Per-vessel daily retention limit. |
| NVessel | vessels | Number of vessels operating in the fleet; scales fleet-wide effort. |
| QuotaMode | — | How the annual quota is set: rate (constant exploitation rate applied to current biomass) or a biomass-linked harvest control rule (Eqs 23a–b). |
| HarvestRate | proportion yr^-1^ | Exploitation rate applied to current target biomass when QuotaMode = rate (Eq 23a). |
| QuotaMin | mt yr^-1^ | Lower clamp on the computed quota (Eq 23b); 0 disables. |
| QuotaMax | mt yr^-1^ | Upper clamp on the computed quota (Eq 23b); 0 disables. |
| Blim | mt | Limit reference biomass; quota is throttled to zero at or below this level (Eq 23b). |
| Bthr | mt | Threshold reference biomass above which the full quota applies (Eq 23b). |

**Table K. Fishery configuration - King Mackerel simulation fleets.**

*Source file: fishery.csv*

Five fleets were configured: one recreational and four commercial, spanning logistic, knife-edge and dome-shaped selectivity.

| **Parameter** | **Rec:**  **Gulfwide** | **Comm:**  **Western_Zone** | **Comm:**  **Northern_Zone** | **Comm:**  **Southern_HookLine** | **Comm:**  **Southern_Gillnet** |
| --- | --- | --- | --- | --- | --- |
| Species | King Mackerel | King Mackerel | King Mackerel | King Mackerel | King Mackerel |
| Role | target | target | target | target | target |
| SelectivityType | logistic | logistic | logistic | knife_edge | dome_shaped |
| L50 | 650 | 700 | 750 | 800 | 610 |
| Slope | 0.015 | 0.02 | 0.02 | 0 | 0.05 |
| L50_2 | 0 | 0 | 0 | 0 | 900 |
| Slope_2 | 0 | 0 | 0 | 0 | 0.02 |
| Quota | 1000 | 580 | 261 | 304 | 304 |
| StartDay | 0 | 0 | 0 | 0 | 21 |
| EndDay | 365 | 365 | 365 | 365 | 365 |
| XMin | -98 | -98 | -87.5 | -87.5 | -84 |
| XMax | -82 | -87.5 | -84 | -82 | -82 |
| YMin | 24 | 25 | 28 | 24 | 24 |
| YMax | 31 | 30 | 30.5 | 28 | 26 |
| ZMin | 0 | 20 | 20 | 20 | 10 |
| ZMax | 150 | 200 | 200 | 200 | 100 |
| SlotMin | 610 | 610 | 610 | 610 | 610 |
| SlotMax | 10000 | 10000 | 10000 | 10000 | 10000 |
| BagLimit | 3 | 6 | 6 | 6 | 230 |
| NVessel | 1000 | 50 | 50 | 50 | 20 |
| QuotaMode | rate | rate | rate | rate | rate |
| HarvestRate | 0.2 | 0.05 | 0.05 | 0.05 | 0.05 |
| QuotaMin | 0 | 0 | 0 | 0 | 0 |
| QuotaMax | 0 | 0 | 0 | 0 | 0 |
| Blim | 0 | 0 | 0 | 0 | 0 |
| Bthr | 0 | 0 | 0 | 0 | 0 |

**Table L. Reproduction seasonality.**

*Source file: reproduction.csv*

Monthly multiplier (*seasonₘᵤₗₜ* in Eq 28) scaling the conversion of accumulated reproductive energy into eggs. Values are dimensionless, 0 = no spawning, 1 = peak spawning. Rows are condensed in this table for brevity, but the CSV file uploaded must have one row per month.

| **Month** | **Multiplier** |
| --- | --- |
| Jan-Mar | 0.01 |
| Apr | 0.15 |
| May-Oct | 1 |
| Nov | 0.5 |
| Dec | 0.01 |

**Table M. Resource growth seasonality.**

*Source file: growth_seasonality.csv*

Monthly multiplier applied to each resource group’s intrinsic growth rate r before the logistic update (Eq 25). Values are dimensionless; 1.0 denotes the annual baseline rate.

| **Species** | **January** | **Feb.** | **March** | **April** | **May** | **June** | **July** | **August** | **Sept.** | **October** | **Nov.** | **Dec.** |
| --- | --- | --- | --- | --- | --- | --- | --- | --- | --- | --- | --- | --- |
| Copepods & Small Zoop. | 0.8 | 0.9 | 1.2 | 1.5 | 1.4 | 1.2 | 1.1 | 1.0 | 1.0 | 0.9 | 0.8 | 0.7 |
| Krill & Euphausiids | 0.7 | 0.8 | 1.1 | 1.4 | 1.5 | 1.3 | 1.1 | 1.0 | 0.9 | 0.8 | 0.7 | 0.7 |
| Gelatinous Zooplankton | 0.6 | 0.7 | 0.9 | 1.2 | 1.5 | 1.6 | 1.5 | 1.3 | 1.1 | 0.8 | 0.6 | 0.5 |
| Anchovy-Sardine Guild | 0.9 | 0.9 | 1.0 | 1.2 | 1.3 | 1.2 | 1.1 | 1.1 | 1.0 | 1.0 | 0.9 | 0.9 |
| Small pelagic fish | 0.9 | 0.9 | 1.0 | 1.1 | 1.2 | 1.2 | 1.1 | 1.1 | 1.1 | 1.0 | 0.9 | 0.9 |
| Pelagic Invertebrates | 0.8 | 0.8 | 1.0 | 1.3 | 1.4 | 1.3 | 1.2 | 1.1 | 1.0 | 0.9 | 0.8 | 0.8 |
| Spanish Mackerel | 1.0 | 1.0 | 1.0 | 1.0 | 1.1 | 1.1 | 1.1 | 1.1 | 1.0 | 1.0 | 1.0 | 1.0 |
| Other Mackerel | 1.0 | 1.0 | 1.0 | 1.0 | 1.1 | 1.1 | 1.1 | 1.1 | 1.0 | 1.0 | 1.0 | 1.0 |
| Cephalopods | 0.8 | 0.9 | 1.1 | 1.2 | 1.3 | 1.3 | 1.2 | 1.1 | 1.1 | 1.0 | 0.9 | 0.8 |
| Small tunas | 1.0 | 1.0 | 1.0 | 1.0 | 1.1 | 1.1 | 1.1 | 1.1 | 1.0 | 1.0 | 1.0 | 1.0 |
| Pelagic fishes | 0.9 | 0.9 | 1.0 | 1.1 | 1.2 | 1.2 | 1.1 | 1.1 | 1.1 | 1.0 | 0.9 | 0.9 |
| Medium-sized fishes | 0.9 | 0.9 | 1.0 | 1.1 | 1.2 | 1.2 | 1.1 | 1.1 | 1.1 | 1.0 | 0.9 | 0.9 |
| Other Large Sharks | 0.9 | 0.9 | 1.0 | 1.1 | 1.1 | 1.1 | 1.1 | 1.1 | 1.0 | 1.0 | 0.9 | 0.9 |
| Large coastal pelagics | 0.9 | 0.9 | 1.0 | 1.1 | 1.1 | 1.1 | 1.1 | 1.1 | 1.0 | 1.0 | 0.9 | 0.9 |
| Cetaceans | 0.9 | 0.9 | 1.0 | 1.1 | 1.1 | 1.1 | 1.1 | 1.1 | 1.0 | 1.0 | 0.9 | 0.9 |
| Benthic Crustaceans | 0.8 | 0.8 | 1.0 | 1.2 | 1.4 | 1.4 | 1.3 | 1.2 | 1.1 | 1.0 | 0.9 | 0.8 |
| Demersal Forage Fishes | 0.9 | 0.9 | 1.0 | 1.1 | 1.2 | 1.2 | 1.1 | 1.1 | 1.1 | 1.0 | 0.9 | 0.9 |

**Table N. Recruitment environment parameters.**

*Source file: recruitment_env.csv*

Controls the environmental and density-dependent modulation of recruitment success.

| **Parameter** | **Value** | **Units** | **Description** |
| --- | --- | --- | --- |
| Species | King Mackerel | — | Species to which the recruitment forcing applies. |
| T_opt | 27.0 | °C | Temperature at which the environmental recruitment multiplier is maximal. |
| T_sd | 3 | °C | Standard deviation of the Gaussian temperature response; larger values broaden the thermal window. |
| sigma_R | 0.5 | dimensionless | Standard deviation of log-normal recruitment variability (recruitment noise). |
| dd_halfsat | 78500000 | g | Spawner-biomass half-saturation constant for density-dependent recruitment; survival halves at this local spawning biomass. |

**Fig A.** Stock-recruitment relationship produced from the King Mackerel simulation with a steepness value of 0.45. Although the latest assessment utilized a steepness of 0.99, but referenced a high degree of uncertainty and biologically derived values of 0.7 (0.59-0.81; SEDAR 38). Given the typical uncertainty in a steepness value and the high degree of interannual variance uncovered in this simulation, we assert that *SwimmingIndividuals* can produce stock-recruitment relationship values, but express caution in their subsequent uptake in other population dynamics models.
