# Supplementary material for: SwimmingIndividuals: A high-performance agent-based model for marine ecosystems
Source: PLoS Comput Biol. 2026 Nov 6;22(11):e1013610. doi: 10.1371/journal.pcbi.1013610 (PMC13766604; doi:10.1371/journal.pcbi.1013610)
Supplement: Supplementary file 2 [file pcbi.1013610.s003.docx]

**Response to Reviewers**

We thank the Academic Editor and the three reviewers for their thorough and constructive evaluation of our manuscript. We respond to each comment individually below. Throughout, the editor and reviewer comments are shown in black and our responses in blue. We are grateful for the feedback, which substantially improved the manuscript, and we would be glad to make any of the outstanding changes noted above on request.

**Editor Comments**

1) We ask that a manuscript source file is provided at Revision. Please upload your manuscript file as a .doc, .docx, .rtf or .tex. If you are providing a .tex file, please upload it under the item type u2018LaTeX Source Fileu2019 and leave your .pdf version as the item type u2018Manuscriptu2019.

The revised document has been uploaded as a .docx file.

2) Please upload all main figures as separate Figure files in .tif or .eps format. For more information about how to convert and format your figure files please see our guidelines: 
[https://journals.plos.org/ploscompbiol/s/figures](https://urldefense.com/v3/__https:/journals.plos.org/ploscompbiol/s/figures__;!!KVu0SnhVq1hAFvslES2Y!LF0OagIMGVII2BKO4NF3MpD1Hsla_6ApOBjAuso-wZi-60dC_O5NuCDsYpqBq1IoBoyH2zPUt-mFfo22ug$)

All figures have been uploaded as separate .tif files.

3) We have noticed that you have uploaded Supporting Information files, but you have not included a list of legends. Please add a full list of legends for your Supporting Information files after the references list.

A full List of Legends file has been created and uploaded as a .docx file.

4) We are unable to open the following Supporting Information file: SI_Submission.zip. Please kindly revise as necessary and re-upload.

The Supporting Information has been updated and a .docx file has been uploaded.

5) Please amend your detailed Financial Disclosure statement. This is published with the article. It must therefore be completed in full sentences and contain the exact wording you wish to be published.
- State the initials, alongside each funding source, of each author to receive each grant. For example: "This work was supported by the National Institutes of Health (####### to AM; ###### to CJ) and the National Science Foundation (###### to AM)."
- State what role the funders took in the study. If the funders had no role in your study, please state: "The funders had no role in study design, data collection and analysis, decision to publish, or preparation of the manuscript.".
If you did not receive any funding for this study, please simply state: u201cThe authors received no specific funding for this work.u201d

The Financial Disclosure statement has been amended in the submission portal to abide by the requirements of PLOS Computational Biology

6) Figure 1: Please confirm whether you drew the images / clip-art within the figure panels by hand. If you did not draw the images, please provide (a) a link to the source of the images or icons and their license / terms of use; or (b) written permission from the copyright holder to publish the images or icons under our CC BY 4.0 license. Alternatively, you may replace the images with open source alternatives. See these open source resources you may use to replace images / clip-art:
- [https://commons.wikimedia.org](https://urldefense.com/v3/__https:/track.editorialmanager.com/CL0/https:*2F*2Fcommons.wikimedia.org*2F/1/010f019d3f05b9ce-9ba8aa72-9a2b-470a-bc68-b79129530f81-000000/DOvgN8THszA8btqaJG6bdu-RSyUNlPOd4nttK4mDq8k=256__;JSUl!!KVu0SnhVq1hAFvslES2Y!LF0OagIMGVII2BKO4NF3MpD1Hsla_6ApOBjAuso-wZi-60dC_O5NuCDsYpqBq1IoBoyH2zPUt-m_a8Jgiw$)
- [https://openclipart.org/](https://urldefense.com/v3/__https:/track.editorialmanager.com/CL0/https:*2F*2Fopenclipart.org*2F/1/010f019d3f05b9ce-9ba8aa72-9a2b-470a-bc68-b79129530f81-000000/M1qSxRtmPwUtD1L6Hd9HUWUSwkX8yGIvdubYkspWeJ0=256__;JSUl!!KVu0SnhVq1hAFvslES2Y!LF0OagIMGVII2BKO4NF3MpD1Hsla_6ApOBjAuso-wZi-60dC_O5NuCDsYpqBq1IoBoyH2zPUt-liBr6jKg$)

All schematic icons in Figure 1 were created by the authors. We have added an explicit statement to the Figure 1 caption: “All schematic icons in Figure 1 were created by the authors and contain no third-party copyrighted elements.”

**Reviewer 1**

Reviewer #1: The manuscript introduces SwimmingIndividuals, an open-source, high-performance agent-based marine ecosystem modeling framework implemented in Julia with a hybrid CPU/GPU design, emphasizing mechanistic submodels (physics-based visual predation, adaptive movement such as DVM, and a flexible bioenergetics engine) and demonstrating emergent patterns across several case studies. The paper is generally readable, but key claims about biological realism, scalability, and validation would benefit from sharper definition and more audit-ready methodological detail.

Thank you for the review. We have addressed each comment individually and have generally applied each suggested revision. We did not utilize the same deep-learning techniques citations recommended by the Reviewer, as other deep learning/ecology-focused references were more appropriate for this particular topic.

1.The manuscript should more explicitly articulate its non-substitutable advances over existing ecological ABM/IBM frameworks, i.e., which specific mechanistic modules and/or software-engineering design choices deliver a substantive improvement rather than an incremental integration.

We agree. We added a dedicated *Comparison to Existing ABM and IBM Frameworks* section that contrasts SwimmingIndividuals with Ecospace, Atlantis, OSMOSE, NetLogo-based IBMs, SEAPODYM, and Ichthyop, identifying this model differs in functional coverage and process realism. Throughout the manuscript, we explicitly state three specific technical advances that, to our knowledge, are not jointly available in existing ABM frameworks: (i) a physics-based visual-detection model in which the perception radius scales dynamically with body size, depth, and ambient irradiance rather than a fixed radius; (ii) a bioenergetics engine that accommodates three distinct metabolic architectures (ectothermic fish, endothermic cetaceans, deep-sea organisms) simultaneously within one framework; and (iii) a hybrid CPU/GPU architecture (Julia, KernelAbstractions.jl) enabling ecosystem-scale populations of superindividuals. We believe these adjustments speak to the novelty of the framework.

2.Key mechanistic components—such as vision-based predation, handling time, gut-fullness–limited ingestion, and the mathematical specification/parameter provenance of movement planning and habitat selection—should be stated more concretely; otherwise it remains unclear whether the reported outcomes are mechanism-driven or primarily parameter-driven.

We added a new *Governing Equations* section that specifies every sub-model mathematically (Eqs 1–28). Visual predation is derived from light-attenuation theory following Aksnes and Giske in the new “Visual Detection and Predator Perception” subsection (Beer’s Law, Eq 1; visual range, Eq 2). The three-step predation sequence now details the per-timestep stomach ceiling, the handling-time limit, and the gut-fullness–dependent feed trigger, so that ingestion is jointly limited by prey availability, travel time, handling time, and satiation rather than a prescribed maximum-consumption parameter. Movement and habitat selection are specified in the new “Habitat Quality and Habitat-Seeking Movement” section (Eqs 18a–d) and the A* pathfinding description. Parameter provenance for these routines is given in the new *Model Inputs and Configuration* section and Supplemental Tables S1–S14, which list each parameter with its value for these simulations and units.

3.The statistical meaning and implications of using super-individuals (where one agent represents many individuals) require clarification, including the weighting scheme and how aggregation affects event rates and density-dependent processes (e.g., mortality, feeding, reproduction).

We expanded the superindividual description in the *Design and Implementation* section. Each agent explicitly carries an *abundance* field (the number of identical individuals it represents) together with two biomass fields, *biomass_ind_* and *biomass_school_*. We clarify that individual-scale processes (e.g., respiration) are computed per individual, whereas population-scale processes (predation and fisheries mortality) operate on agent-level biomass and are scaled by *abundance*, so that event rates are correctly weighted. Density dependence is now made explicit in the reproduction/recruitment formulation (Eq 28 and the recruitment survival terms), where per-egg survival declines with local conspecific spawner biomass, producing an emergent Beverton–Holt compensatory relationship from local crowding rather than an imposed stock-level relationship.

4.The dependence of results on inputs and defaults should be made more transparent. Because the case studies rely on environmental grids, habitat carrying-capacity fields, resource-group definitions, and seasonal forcing/growth settings, the authors should specify the default configuration, the key parameter files, and the minimal input bundle required for full reproducibility.

The new *Model Inputs and Configuration* section describes the full input structure (i.e., the master manifest (files.csv), simulation parameters (params.csv), focal and resource traits (traits.csv, resource_traits.csv), environmental preferences (envi_pref.csv), the environmental-raster catalogue (environment.xml), the fisheries table (fisheries.csv), and the optional recruitment_env.csv). Every parameter, its default value, and its units are tabulated in the Supplemental Material (S1 Text; Tables S1–S14) for the most inclusive (King Mackerel) case study. The complete input bundles for all three case studies are provided in the public GitHub repository (github.com/mattwoodstock/SwimmingIndividuals), one folder per scenario.

5.While the Introduction discusses limitations of traditional models and motivates ABMs, the manuscript provides an insufficiently rigorous comparison to closely related end-to-end IBM/ABM toolkits in terms of functional coverage, scalability, and process realism, which weakens the “state-of-the-art software” positioning; this comparison should be strengthened and made more concrete.

This is addressed by the new *Comparison to Existing ABM and IBM Frameworks* section (see also our response to Comment 1). For each of Ecospace, Atlantis, OSMOSE, NetLogo IBMs, SEAPODYM, and Ichthyop we state what the framework resolves and what it does not (e.g., pool-based vs. individual resolution, stochastic accessibility vs. mechanistic visual detection, presence or absence of an explicit energy budget or 3-D depth structure), and we position SwimmingIndividuals against them on process realism and scalability. Scalability is further supported by the new performance discussion and the hybrid CPU/GPU architecture description.

6.The authors propose an effective strategy. In future work, widely developed deep learning techniques and their application to more complex scenarios (DOI: 10.1145/3664647.3681673, DOI: 10.1109/JBHI.2024.3357979) could be further explored.

We added a “*Future Research Directions”* section that, in part, connects the framework to emerging deep-learning techniques for ecological parameterization and scenario planning, framed around building open-source “digital twins” for predictive marine science (see *Future Research Directions*; citing Tzachor et al. 2023, reference 53). We did not, however, incorporate the two specific references cited by the reviewer (DOIs 10.1145/3664647.3681673 and 10.1109/JBHI.2024.3357979); only the digital-twins reference (53) was added. If the reviewer and editor would like these two works cited specifically, we are glad to add them, but we do not feel that the papers entitled “Discovering Consensus Regions for Interpretable Identification of RNA N6-Methyladenosine Modification Sites via Graph Contrastive Clustering” and “Mitigating World Biases: A Multimodal Multi-View Debiasing Framework for Fake News Video Detection” are the most appropriate in this instance.

**Reviewer 2**

This paper is not truly paper in the ordinary sense. It is more an illustration of a computer code. It describes how the programme "SwimmingIndividuals" is constructed with a nice flow chart and gives some information about the population of the King-Mackerel.

Thank you for your review. We have made major revisions to the manuscript that, in our opinion, align this manuscript more to a traditional paper. Specifically, we have greatly expanded the discussion about the framework’s novelty and methods, and then apply three case studies before an extended discussion on the model’s capabilities and limitations.

As the authors write
"SwimmingIndividuals can serve as a testbed for management strategies by simulating the impacts
of fishing. In the Mackerel scenario, distinct harvest control rules for five different fisheries
resulted in different rates of quota attainment (Fig 8A) and variable mean lengths of caught fish
The model tracks the resulting time-varying fishing mortality for specific size classeand can map its spatial distribution, providing a tool to evaluate the efficacy of time area closures."

Although this tool may be valuable, I would have liked to see a clear comparison with either other models, or real data.
I would therefore recommend a rigorous comparison with other results or some other test from which the validity of the model can be infered, to have means to quantify the strength and scientific value of the code.

We added both of these suggestions and have reframed the paper to more accurately describe the results being shown from the case studies. For real-data validation, the *Results* now compare emergent model output against independent empirical benchmarks: simulated mass-specific metabolic costs (Fig 6B) fall within the published standard-metabolic-rate bounds for pelagic scombroids (refs 42, 43); emergent adult daily rations (Fig 6C) match empirical stomach-content estimates for coastal migratory pelagics (ref 44); and the emergent length-at-age growth curve (Fig 6D) is compared directly against the von Bertalanffy curve from the SEDAR 38 Gulf of Mexico King Mackerel stock assessment (ref 37). We also report an emergent Beverton–Holt steepness (Supplemental Figure 1). For model-to-model context, the new *Comparison to Existing ABM and IBM Frameworks* section situates SwimmingIndividuals against six established frameworks. Together these give the reader both empirical and model-based reference points for judging validity.

**Reviewer 3**

Reviewer #3: Woodstock et al. present a new agent-based marine ecosystem model. The manuscript is well-written, and the new model is innovative, taking advantage of a novel GPU/CPU architecture in the Julia programming language. The code to run the model is available and well-documented. I commend the authors for this work, developing and presenting a new marine ecosystem model in the way they have is a long process. I have one major comment, and several minor comments.

Thank you for your review. We have addressed each comment below.

Major comment:
I was missing an overview of the governing equations of the model. The model code and workflow is clearly documented, but the model itself (in terms of its equations) is missing. Figure 1 does a good job at providing an overview of the structure of the model, but associated equations should at least be included in a supplementary.

We fully agree. We added a comprehensive *Governing Equations* section to the main text (rather than only the supplement), presenting the complete mathematical specification of the model across nine subsections and Equations 1–28 — covering visual detection, predation and ingestion, bioenergetics and respiration, growth and reproduction, habitat quality, vertical movement, resource dynamics, mortality, and recruitment. The full parameterization accompanying these equations is provided in the Supplemental Material for the most inclusive (King Mackerel) case study (Tables S1–S14).

Minor comments:
1) For the examples in the manuscript, where are the parameters taken from?

The source of every literature-derived parameter is now given explicitly in the Supplemental Material (S1 Text; Tables S1–S14), where each entry lists its value, units, and role, and the bioenergetic and life-history coefficients are tied to their literature sources in the *Governing Equations* section. The King Mackerel case-study parameters are drawn from the SEDAR 38 stock assessment (ref 37) and the associated species literature cited therein. Note, that the resource species were designed to represented functional groups that increase in size, but are not representative of specific species.

2) Font size in most figures is too small. For example, text on the arrows in figure 1 is unreadable without zooming in. What are the time slices in figure 7b? Also, in Figure 4 state in caption the region if the Gulf of Mexico.

We revised the figures and figure captions for clarity: the Figure 1 caption now defines the energy-flux abbreviations (A, R, SDA, U, F) that the arrows denote, and the Figure 7 caption now specifies that panel B shows the size-frequency distribution at four distinct multi-decadal time slices. Figure 4 now states the region is the Gulf of Mexico, although that is appliable to each case study.

3) Great to see a model that accounts for environmental conditions in an organism's visual range. Seeing how this is done an equation would be even better.

The new “Visual Detection and Predator Perception” subsection derives the visual range mechanistically following Aksnes and Giske: surface irradiance follows a Gaussian diel curve, light is attenuated with depth by Beer’s Law (Eq 1), and the realized visual range is given by Eq 2, bounded by a body-length–scaled maximum (r_max = 30L). The relevant coefficients and their sources are listed in the Supplemental Material.

4) Could DVM not be included in habitat quality rather than a manual override? Since it's most likely triggered by an avoidance of predators? I was missing a summary of what determines habitat quality.

The second part is fully addressed: we added a new section, *Habitat Quality and Habitat-Seeking Movement* (Section 5, Eqs 18a–d), which summarizes how the multi-variable habitat-capacity score is computed from each species’ environmental preference bounds and how agents move up the resulting gradient via A* pathfinding. Regarding the first part, we clarified in the *Diel Vertical Migration and Behavioral Archetypes* section that DVM and diving are currently implemented as archetype-driven vertical overrides, and we explicitly discuss making vertical position an emergent, state-dependent decision (balancing light-dependent predation risk against prey encounter) as a priority for future development in the *Future Research Directions*. We addressed this suggestion by explanation and as a stated future direction rather than by re-implementing the mechanism in this version (v1.0), which we judged beyond the scope of the current release.
