# Supplementary material for: SwimmingIndividuals: A high-performance agent-based model for marine ecosystems
Source: PLoS Comput Biol. 2026 Nov 6;22(11):e1013610. doi: 10.1371/journal.pcbi.1013610 (PMC13766604; doi:10.1371/journal.pcbi.1013610)
Supplement: Peer review history

# Peer review: Decision Letter 0

**DOI:** 10.1371/journal.pcbi.1013610.r001

**Authors:** Zhanshan (Sam) Ma, Tobias Bollenbach

30 Mar 2026

SwimmingIndividuals: A High-Performance Agent-Based Model for Marine Ecosystems

PLOS Computational Biology

Dear Dr. Woodstock,

Thank you for submitting your manuscript to PLOS Computational Biology. After careful consideration, we feel that it has merit but does not fully meet PLOS Computational Biology's publication criteria as it currently stands. Therefore, we invite you to submit a revised version of the manuscript that addresses the points raised during the review process.

Please submit your revised manuscript by May 30 2026 11:59PM. If you will need more time than this to complete your revisions, please reply to this message or contact the journal office at ploscompbiol@plos.org. When you're ready to submit your revision, log on to https://www.editorialmanager.com/pcompbiol/ and select the 'Submissions Needing Revision' folder to locate your manuscript file.

Please include the following items when submitting your revised manuscript:

* A letter that responds to each point raised by the editor and reviewer(s). You should upload this letter as a separate file labeled 'Response to Reviewers'. This file does not need to include responses to formatting updates and technical items listed in the 'Journal Requirements' section below.

* A marked-up copy of your manuscript that highlights changes made to the original version. You should upload this as a separate file labeled 'Revised Manuscript with Track Changes'.

* An unmarked version of your revised paper without tracked changes. You should upload this as a separate file labeled 'Manuscript'.

If you would like to make changes to your financial disclosure, competing interests statement, or data availability statement, please make these updates within the submission form at the time of resubmission. Guidelines for resubmitting your figure files are available below the reviewer comments at the end of this letter

We look forward to receiving your revised manuscript.

Kind regards,

Zhanshan (Sam) Ma, Ph.D

Academic Editor

PLOS Computational Biology

Tobias Bollenbach

Section Editor

PLOS Computational Biology

**Journal Requirements:**

1) We ask that a manuscript source file is provided at Revision. Please upload your manuscript file as a .doc, .docx, .rtf or .tex. If you are providing a .tex file, please upload it under the item type u2018LaTeX Source Fileu2019 and leave your .pdf version as the item type u2018Manuscriptu2019.

2) Please upload all main figures as separate Figure files in .tif or .eps format. For more information about how to convert and format your figure files please see our guidelines:

https://journals.plos.org/ploscompbiol/s/figures

3) We have noticed that you have uploaded Supporting Information files, but you have not included a list of legends. Please add a full list of legends for your Supporting Information files after the references list.

4) We are unable to open the following Supporting Information file: SI_Submission.zip. Please kindly revise as necessary and re-upload.

5) Please amend your detailed Financial Disclosure statement. This is published with the article. It must therefore be completed in full sentences and contain the exact wording you wish to be published.

- State the initials, alongside each funding source, of each author to receive each grant. For example: "This work was supported by the National Institutes of Health (####### to AM; ###### to CJ) and the National Science Foundation (###### to AM)."

- State what role the funders took in the study. If the funders had no role in your study, please state: "The funders had no role in study design, data collection and analysis, decision to publish, or preparation of the manuscript.".

If you did not receive any funding for this study, please simply state: u201cThe authors received no specific funding for this work.u201d

6) Figure 1: Please confirm whether you drew the images / clip-art within the figure panels by hand. If you did not draw the images, please provide (a) a link to the source of the images or icons and their license / terms of use; or (b) written permission from the copyright holder to publish the images or icons under our CC BY 4.0 license. Alternatively, you may replace the images with open source alternatives. See these open source resources you may use to replace images / clip-art:

- https://commons.wikimedia.org

- https://openclipart.org/

**Reviewers' comments:**

Reviewer's Responses to Questions

**Comments to the Authors:**

**Please note here if the review is uploaded as an attachment.**

Reviewer #1: The manuscript introduces SwimmingIndividuals, an open-source, high-performance agent-based marine ecosystem modeling framework implemented in Julia with a hybrid CPU/GPU design, emphasizing mechanistic submodels (physics-based visual predation, adaptive movement such as DVM, and a flexible bioenergetics engine) and demonstrating emergent patterns across several case studies. The paper is generally readable, but key claims about biological realism, scalability, and validation would benefit from sharper definition and more audit-ready methodological detail.

1.The manuscript should more explicitly articulate its non-substitutable advances over existing ecological ABM/IBM frameworks, i.e., which specific mechanistic modules and/or software-engineering design choices deliver a substantive improvement rather than an incremental integration.

2.Key mechanistic components—such as vision-based predation, handling time, gut-fullness–limited ingestion, and the mathematical specification/parameter provenance of movement planning and habitat selection—should be stated more concretely; otherwise it remains unclear whether the reported outcomes are mechanism-driven or primarily parameter-driven.

3.The statistical meaning and implications of using super-individuals (where one agent represents many individuals) require clarification, including the weighting scheme and how aggregation affects event rates and density-dependent processes (e.g., mortality, feeding, reproduction).

4.The dependence of results on inputs and defaults should be made more transparent. Because the case studies rely on environmental grids, habitat carrying-capacity fields, resource-group definitions, and seasonal forcing/growth settings, the authors should specify the default configuration, the key parameter files, and the minimal input bundle required for full reproducibility.

5.While the Introduction discusses limitations of traditional models and motivates ABMs, the manuscript provides an insufficiently rigorous comparison to closely related end-to-end IBM/ABM toolkits in terms of functional coverage, scalability, and process realism, which weakens the “state-of-the-art software” positioning; this comparison should be strengthened and made more concrete.

6.The authors propose an effective strategy. In future work, widely developed deep learning techniques and their application to more complex scenarios (DOI: 10.1145/3664647.3681673, DOI: 10.1109/JBHI.2024.3357979) could be further explored.

Reviewer #2: This paper is not truly paper in the ordinary sense. It is more an illustration of a computer code. It describes how the programme "SwimmingIndividuals" is constructed with a nice flow chart and gives some information about the population of the King-Mackerel.

As the authors write

"SwimmingIndividuals can serve as a testbed for management strategies by simulating the impacts

of fishing. In the Mackerel scenario, distinct harvest control rules for five different fisheries

resulted in different rates of quota attainment (Fig 8A) and variable mean lengths of caught fish

The model tracks the resulting time-varying fishing mortality for specific size classeand can map its spatial distribution, providing a tool to evaluate the efficacy of time area closures."

Although this tool may be valuable, I would have liked to see a clear comparison with either other models, or real data.

I would therefore recommend a rigorous comparison with other results or some other test from which the validity of the model can be infered, to have means to quantify the strength and scientific value of the code.

Reviewer #3: Woodstock et al. present a new agent-based marine ecosystem model. The manuscript is well-written, and the new model is innovative, taking advantage of a novel GPU/CPU architecture in the Julia programming language. The code to run the model is available and well-documented. I commend the authors for this work, developing and presenting a new marine ecosystem model in the way they have is a long process. I have one major comment, and several minor comments.

Major comment:

I was missing an overview of the governing equations of the model. The model code and workflow is clearly documented, but the model itself (in terms of its equations) is missing. Figure 1 does a good job at providing an overview of the structure of the model, but associated equations should at least be included in a supplementary.

Minor comments:

1) For the examples in the manuscript, where are the parameters taken from?

2) Font size in most figures is too small. For example, text on the arrows in figure 1 is unreadable without zooming in. What are the time slices in figure 7b? Also, in Figure 4 state in caption the region if the Gulf of Mexico.

3) Great to see a model that accounts for environmental conditions in an organism's visual range. Seeing how this is done an equation would be even better.

4) Could DVM not be included in habitat quality rather than a manual override? Since it's most likely triggered by an avoidance of predators? I was missing a summary of what determines habitat quality.

**********

**Have the authors made all data and (if applicable) computational code underlying the findings in their manuscript fully available?**

The PLOS Data policy requires authors to make all data and code underlying the findings described in their manuscript fully available without restriction, with rare exception (please refer to the Data Availability Statement in the manuscript PDF file). The data and code should be provided as part of the manuscript or its supporting information, or deposited to a public repository. For example, in addition to summary statistics, the data points behind means, medians and variance measures should be available. If there are restrictions on publicly sharing data or code —e.g. participant privacy or use of data from a third party—those must be specified.

Reviewer #1: None

Reviewer #2: Yes

Reviewer #3: Yes

**********

PLOS authors have the option to publish the peer review history of their article (what does this mean?). If published, this will include your full peer review and any attached files.

If you choose “no”, your identity will remain anonymous but your review may still be made public.

**Do you want your identity to be public for this peer review?**  For information about this choice, including consent withdrawal, please see our Privacy Policy.

Reviewer #1: No

Reviewer #2: No

Reviewer #3: No

[NOTE: If reviewer comments were submitted as an attachment file, they will be attached to this email and accessible via the submission site. Please log into your account, locate the manuscript record, and check for the action link "View Attachments". If this link does not appear, there are no attachment files.]

**Figure resubmission:**

**Reproducibility:**

?>

# Author comment: Author response to Decision Letter 1

**DOI:** 10.1371/journal.pcbi.1013610.r002

24 Sep 2026

Attachment Submitted filename: Response_to_Reviewers.docx

# Peer review: Decision Letter 1

**DOI:** 10.1371/journal.pcbi.1013610.r003

**Authors:** Zhanshan (Sam) Ma, Tobias Bollenbach

28 Oct 2026

Dear Dr. Woodstock,

We are pleased to inform you that your manuscript 'SwimmingIndividuals: A High-Performance Agent-Based Model for Marine Ecosystems' has been provisionally accepted for publication in PLOS Computational Biology.

Before your manuscript can be formally accepted you will need to complete some formatting changes, which you will receive in a follow up email. A member of our team will be in touch with a set of requests.

Please note that your manuscript will not be scheduled for publication until you have made the required changes, so a swift response is appreciated.

IMPORTANT: The editorial review process is now complete. PLOS will only permit corrections to spelling, formatting or significant scientific errors from this point onwards. Requests for major changes, or any which affect the scientific understanding of your work, will cause delays to the publication date of your manuscript.

Should you, your institution's press office or the journal office choose to press release your paper, you will automatically be opted out of early publication. We ask that you notify us now if you or your institution is planning to press release the article. All press must be co-ordinated with PLOS.

Thank you again for supporting Open Access publishing; we are looking forward to publishing your work in PLOS Computational Biology.

Best regards,

Zhanshan (Sam) Ma, Ph.D

Academic Editor

PLOS Computational Biology

Tobias Bollenbach

Section Editor

PLOS Computational Biology

***********************************************************

Reviewer's Responses to Questions

**Comments to the Authors:**

**Please note here if the review is uploaded as an attachment.**

Reviewer #1: The manuscript has handled my concerns very well and I recommend accepting it.

Reviewer #2: The paper has been revised to include the comments.

**********

**Have the authors made all data and (if applicable) computational code underlying the findings in their manuscript fully available?**

The PLOS Data policy requires authors to make all data and code underlying the findings described in their manuscript fully available without restriction, with rare exception (please refer to the Data Availability Statement in the manuscript PDF file). The data and code should be provided as part of the manuscript or its supporting information, or deposited to a public repository. For example, in addition to summary statistics, the data points behind means, medians and variance measures should be available. If there are restrictions on publicly sharing data or code —e.g. participant privacy or use of data from a third party—those must be specified.

Reviewer #1: Yes

Reviewer #2: Yes

**********

PLOS authors have the option to publish the peer review history of their article (what does this mean?). If published, this will include your full peer review and any attached files.

If you choose “no”, your identity will remain anonymous but your review may still be made public.

**Do you want your identity to be public for this peer review?**  For information about this choice, including consent withdrawal, please see our Privacy Policy.

Reviewer #1: No

Reviewer #2: No

# Editor report: Acceptance letter

**DOI:** 10.1371/journal.pcbi.1013610.r004

**Authors:** Zhanshan (Sam) Ma, Tobias Bollenbach

PCOMPBIOL-D-25-02046R1

SwimmingIndividuals: A High-Performance Agent-Based Model for Marine Ecosystems

Dear Dr Woodstock,

I am pleased to inform you that your manuscript has been formally accepted for publication in PLOS Computational Biology. Your manuscript is now with our production department and you will be notified of the publication date in due course.

The corresponding author will soon be receiving a typeset proof for review, to ensure errors have not been introduced during production. Please review the PDF proof of your manuscript carefully, as this is the last chance to correct any errors. Please note that major changes, or those which affect the scientific understanding of the work, will likely cause delays to the publication date of your manuscript.

Soon after your final files are uploaded, unless you have opted out, the early version of your manuscript will be published online. The date of the early version will be your article's publication date. The final article will be published to the same URL, and all versions of the paper will be accessible to readers.

For Research, Software, and Methods articles, you will receive an invoice from PLOS for your publication fee after your manuscript has reached the completed accept phase. If you receive an email requesting payment before acceptance or for any other service, this may be a phishing scheme. Learn how to identify phishing emails and protect your accounts at https://explore.plos.org/phishing.

Thank you again for supporting PLOS Computational Biology and open-access publishing. We are looking forward to publishing your work!

With kind regards,

Janani Seenivasan

PLOS Computational Biology | Carlyle House, Carlyle Road, Cambridge CB4 3DN | United Kingdom ploscompbiol@plos.org | Phone +44 (0) 1223-442824 | ploscompbiol.org | @PLOSCompBiol
